# Supplementary material for: Got 15? Try Faculty Development on the Fly: A Snippets Workshop for Microlearning
Source: MedEdPORTAL. 2021 Jun 14;17:11161. doi: 10.15766/mep_2374-8265.11161 (PMC8200375; doi:10.15766/mep_2374-8265.11161)
Supplement: Supplementary file 1 — Snippet Presentation.pptxSession Plan.docxParticipant Email Message.docxSnippet Template.pptxCurated Materials Learning Environment.docxSmall-Group Instructions.docxExample of Completed Snippet.pptxWorkshop Evaluation.docx [file mep_2374-8265.11161-s001.zip › A. Snippet Presentation.pptx]

## Slide 1
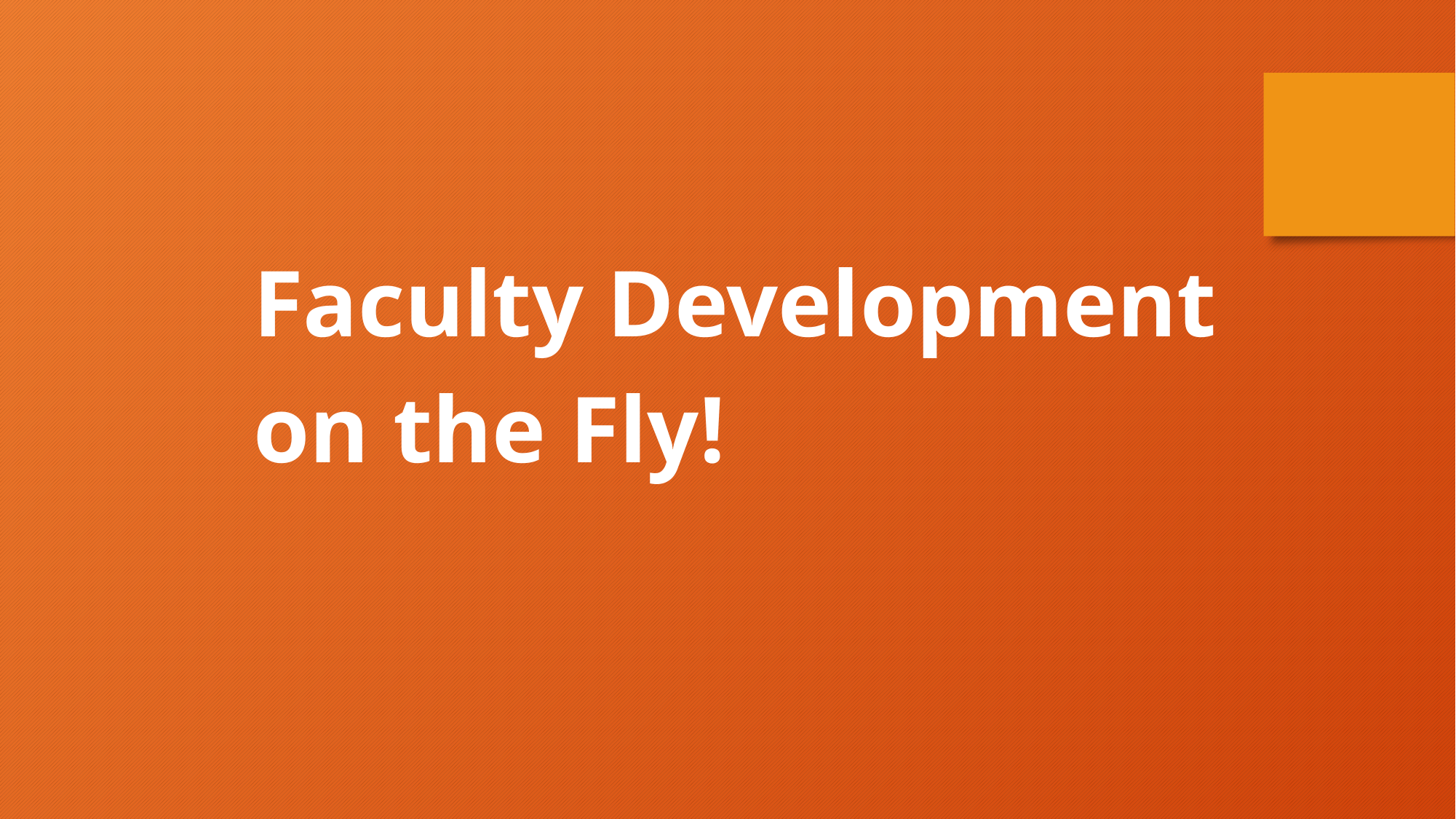

Faculty Development on the Fly!

## Slide 2
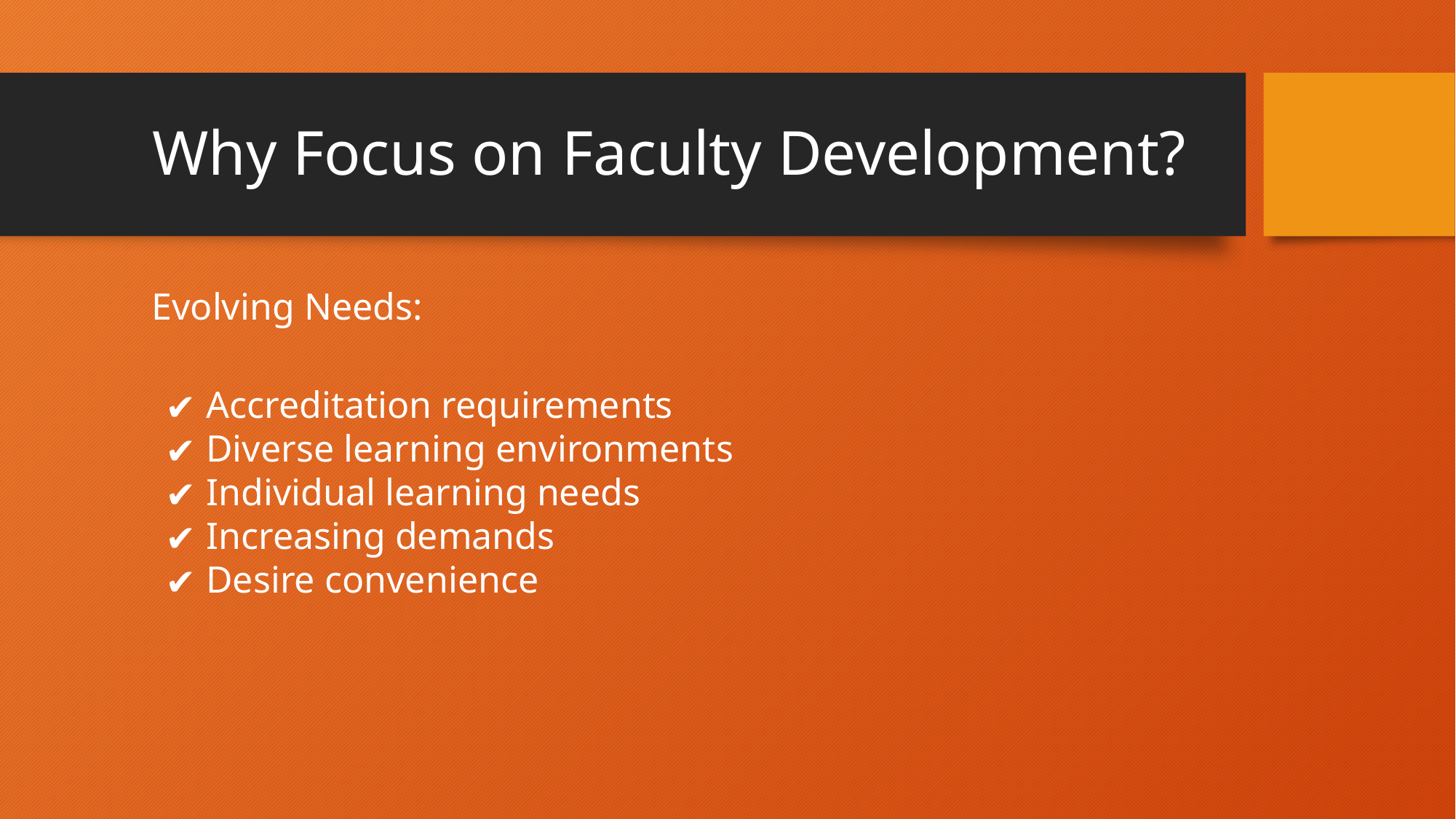

# Why Focus on Faculty Development?
Evolving Needs:
Accreditation requirements
Diverse learning environments
Individual learning needs
Increasing demands
Desire convenience

## Slide 3
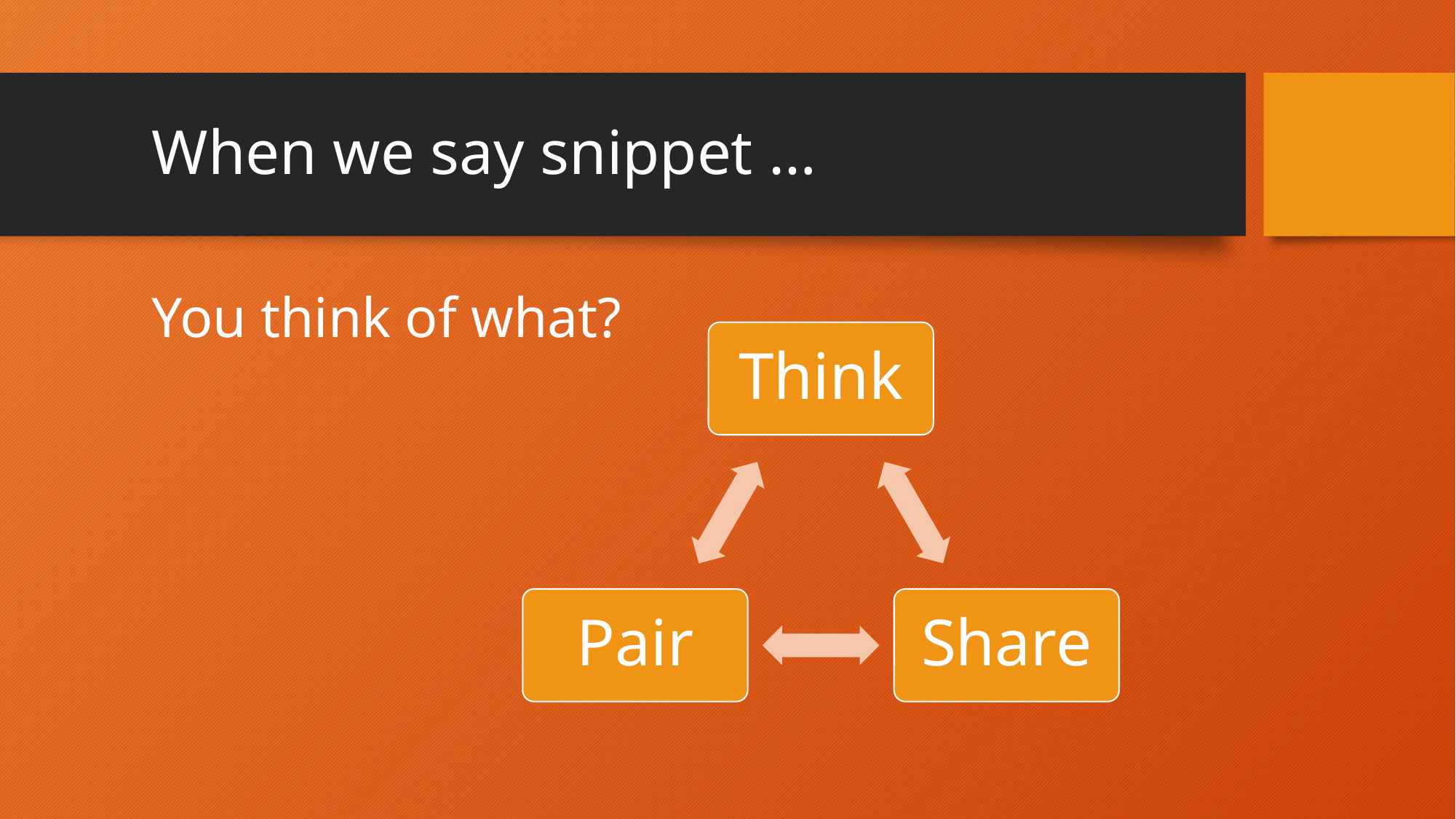

# When we say snippet …
You think of what?
Think
Pair
Share

## Slide 4
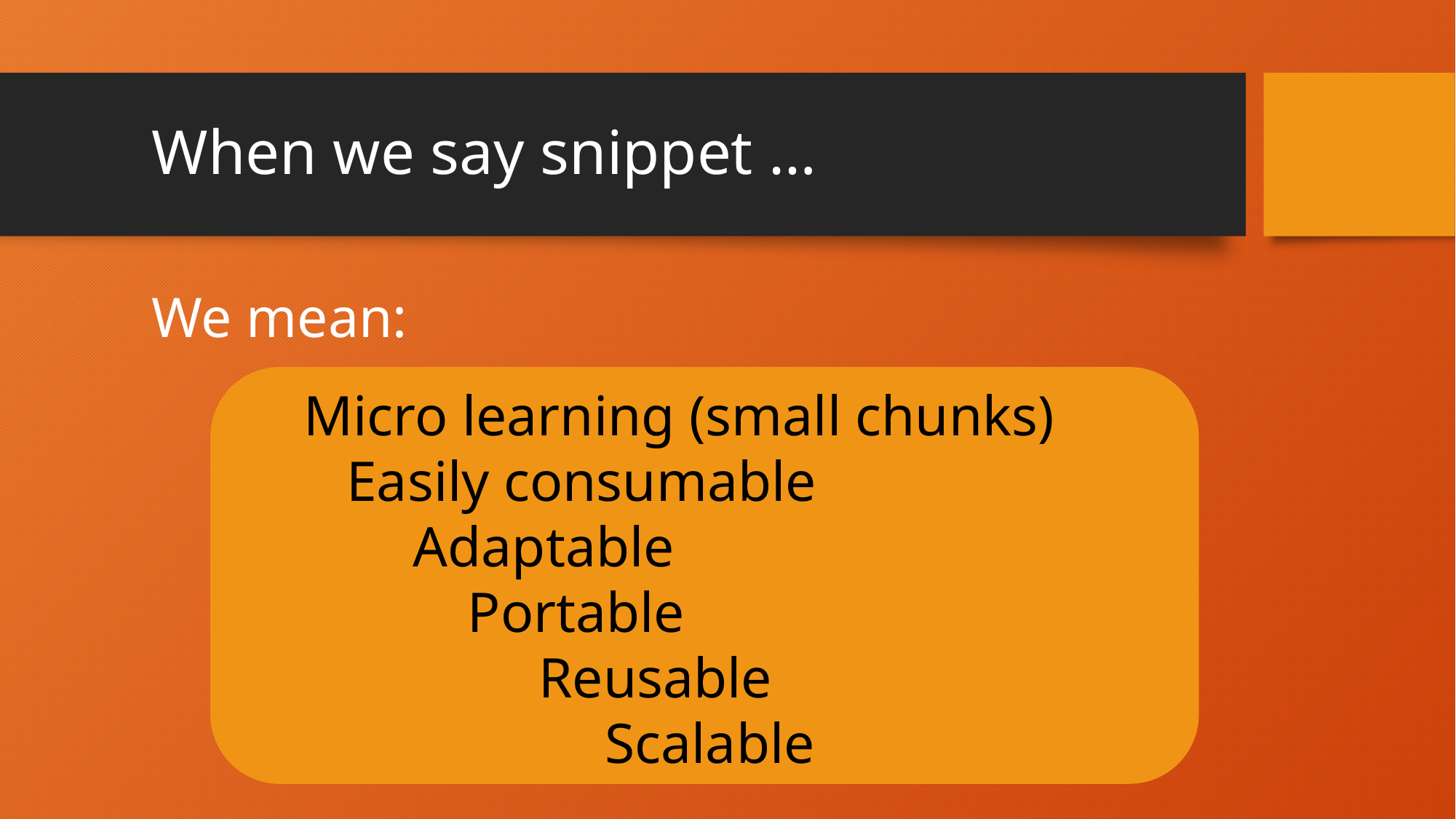

# When we say snippet …
We mean:
Micro learning (small chunks)
 Easily consumable
Adaptable
	Portable
	 Reusable
		 Scalable

## Slide 5
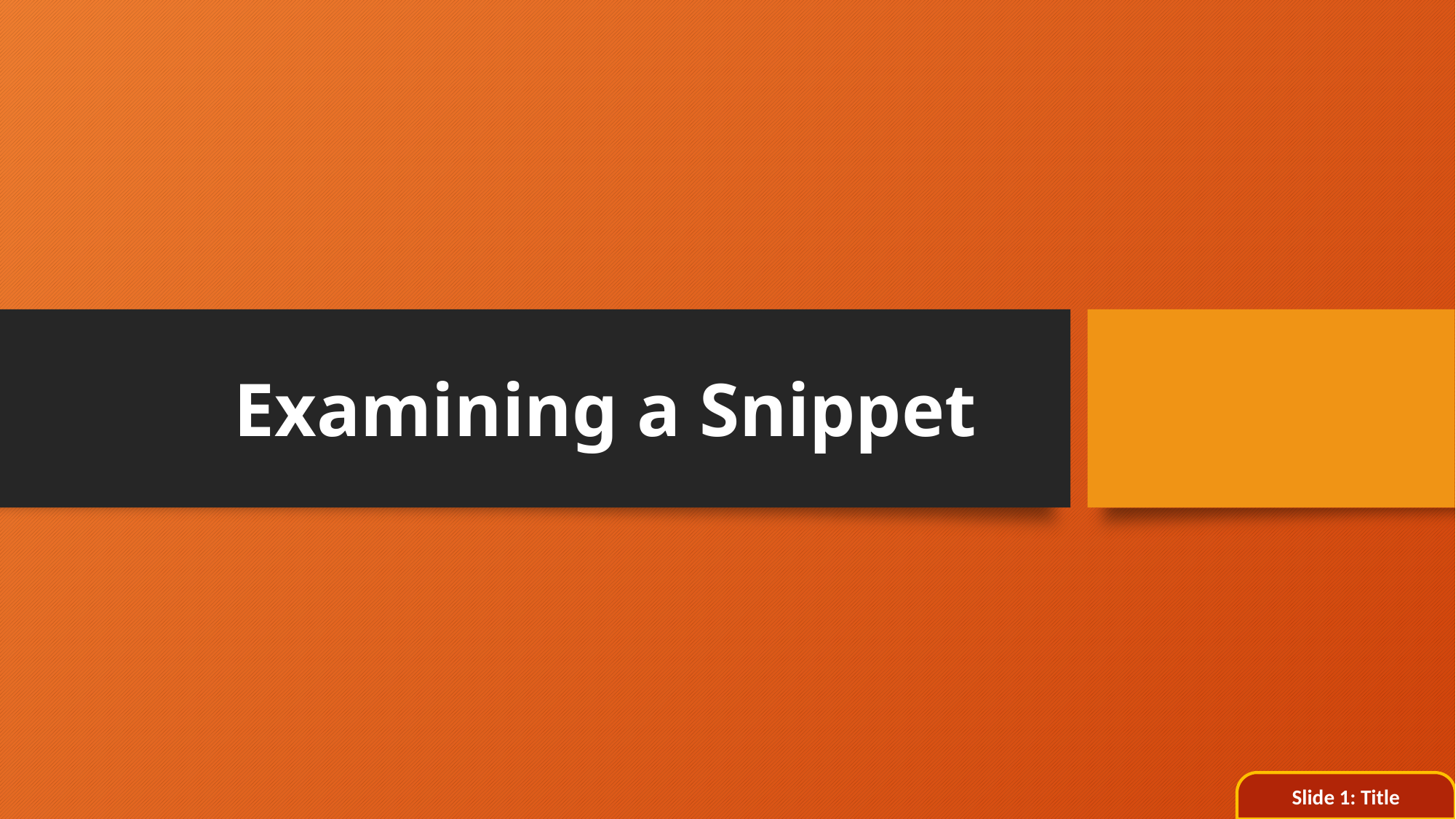

# Examining a Snippet
Slide 1: Title

## Slide 6
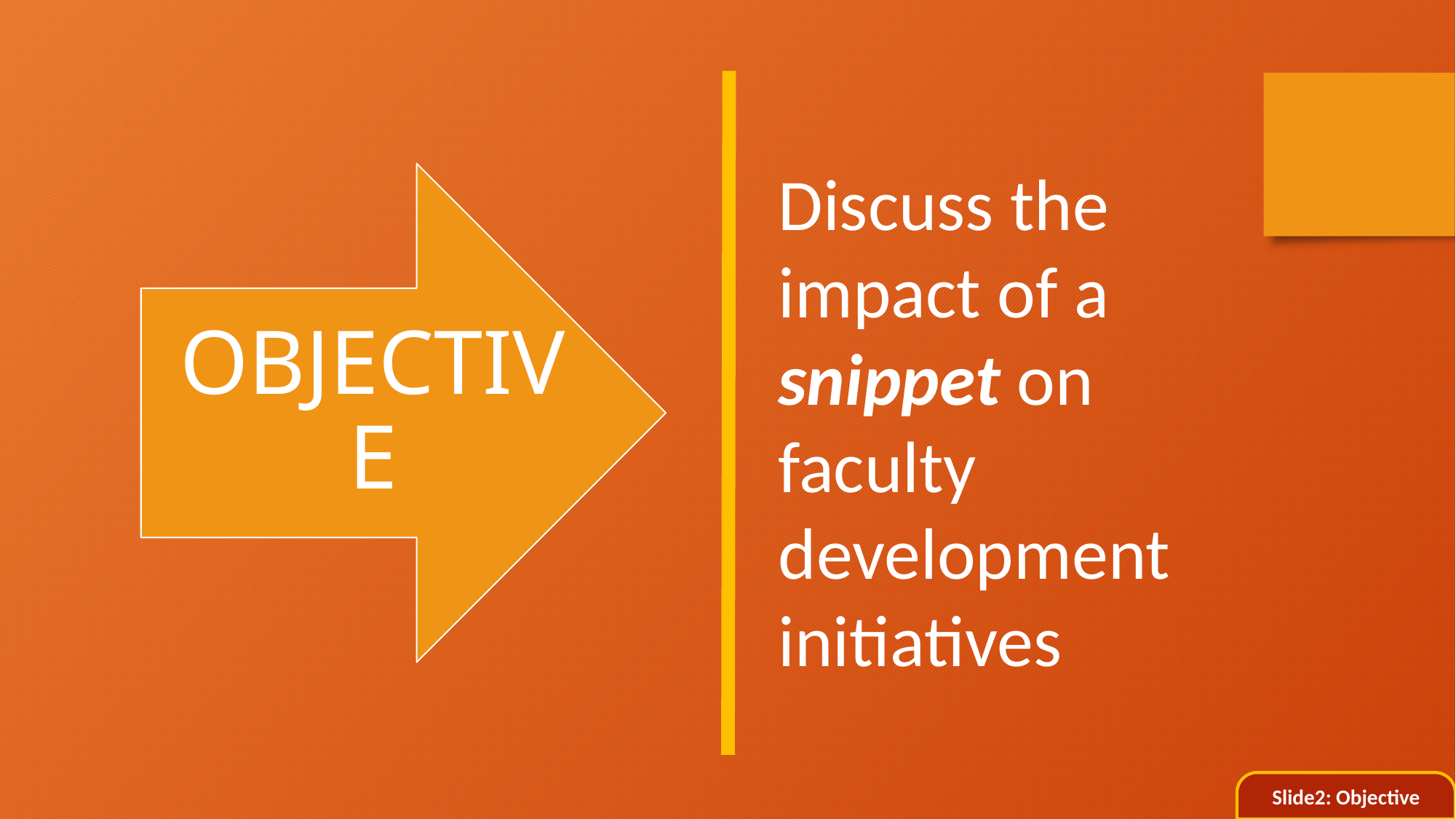

Discuss the impact of a snippet on faculty development initiatives
OBJECTIVE
Slide2: Objective

## Slide 7
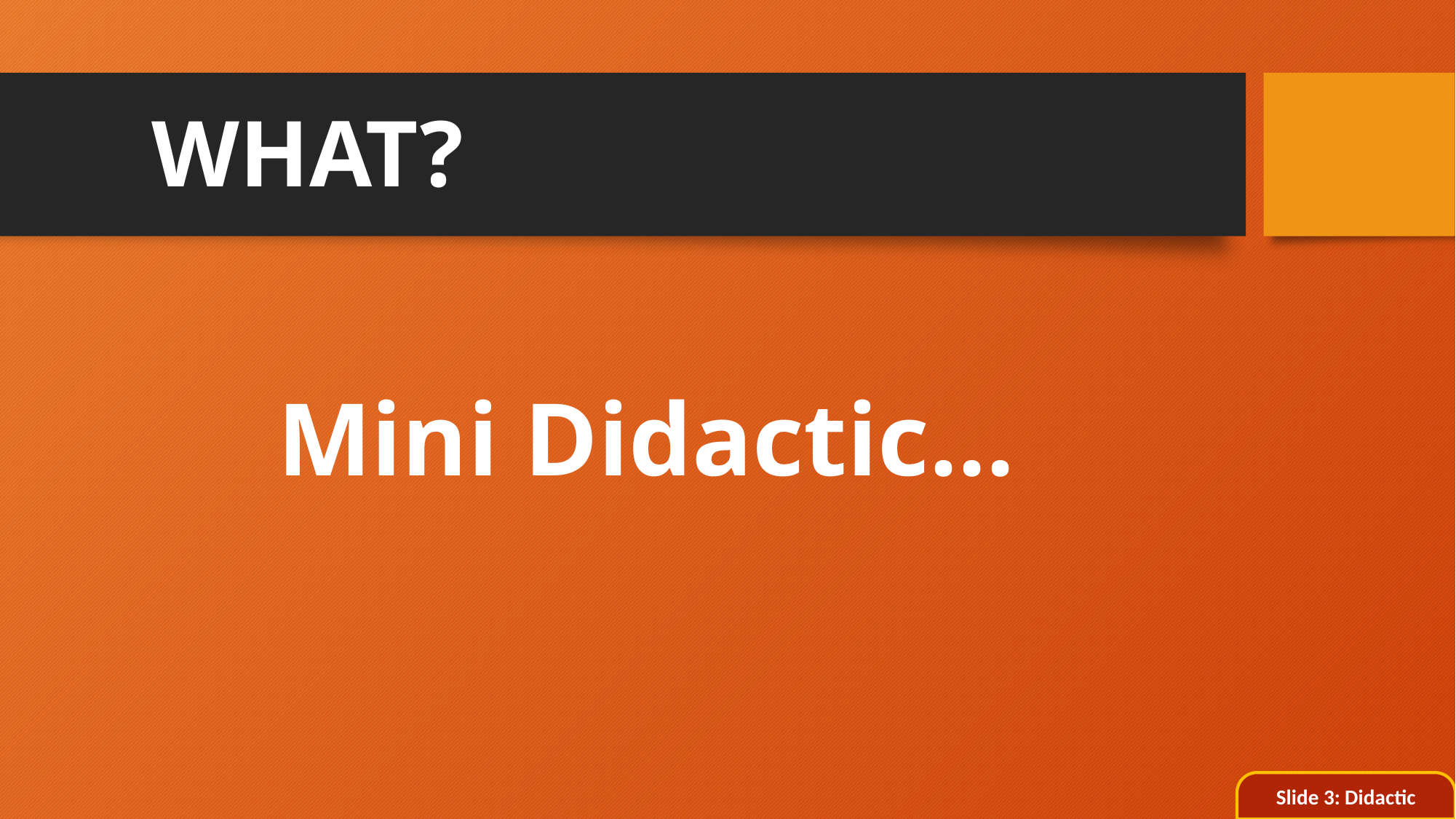

# WHAT?
Mini Didactic...
Slide 3: Didactic

## Slide 8
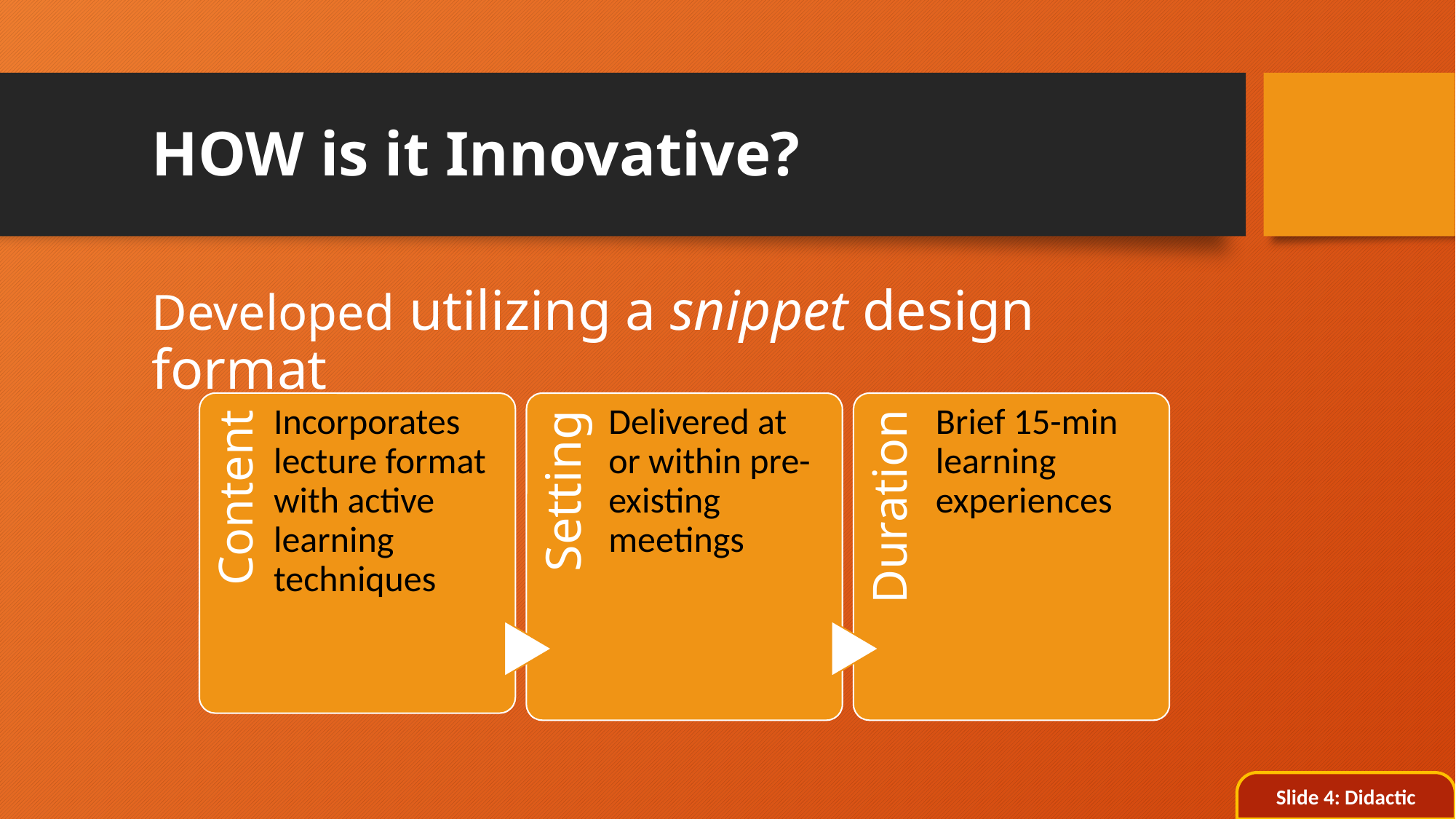

# HOW is it Innovative?
Developed utilizing a snippet design format
Content
Incorporates lecture format with active learning techniques
Delivered at or within pre-existing meetings
Duration
Brief 15-min learning experiences
Setting
Slide 4: Didactic

## Slide 9
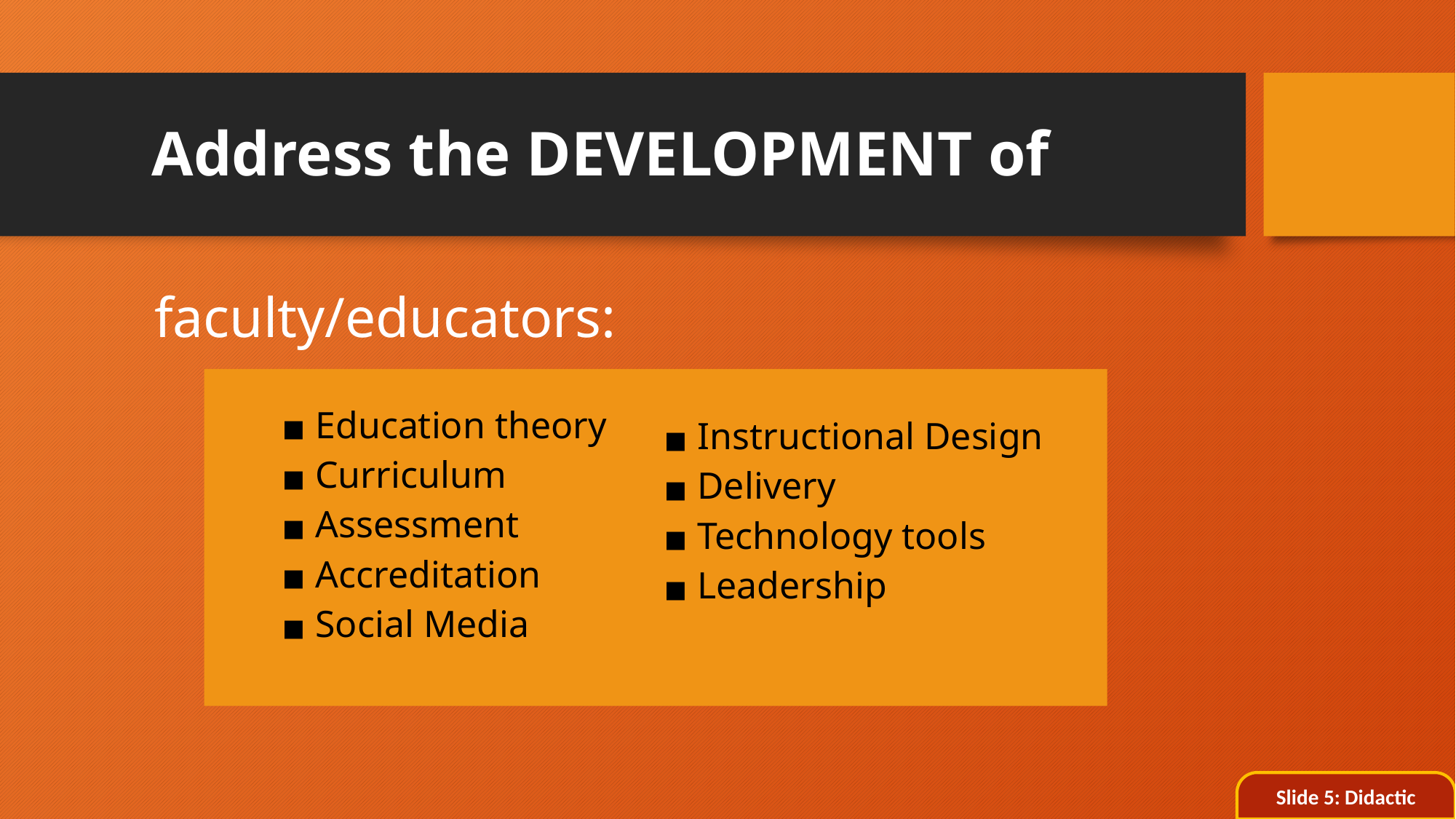

# Address the DEVELOPMENT of
faculty/educators:
Instructional Design
Delivery
Technology tools
Leadership
Education theory
Curriculum
Assessment
Accreditation
Social Media
Slide 5: Didactic

## Slide 10
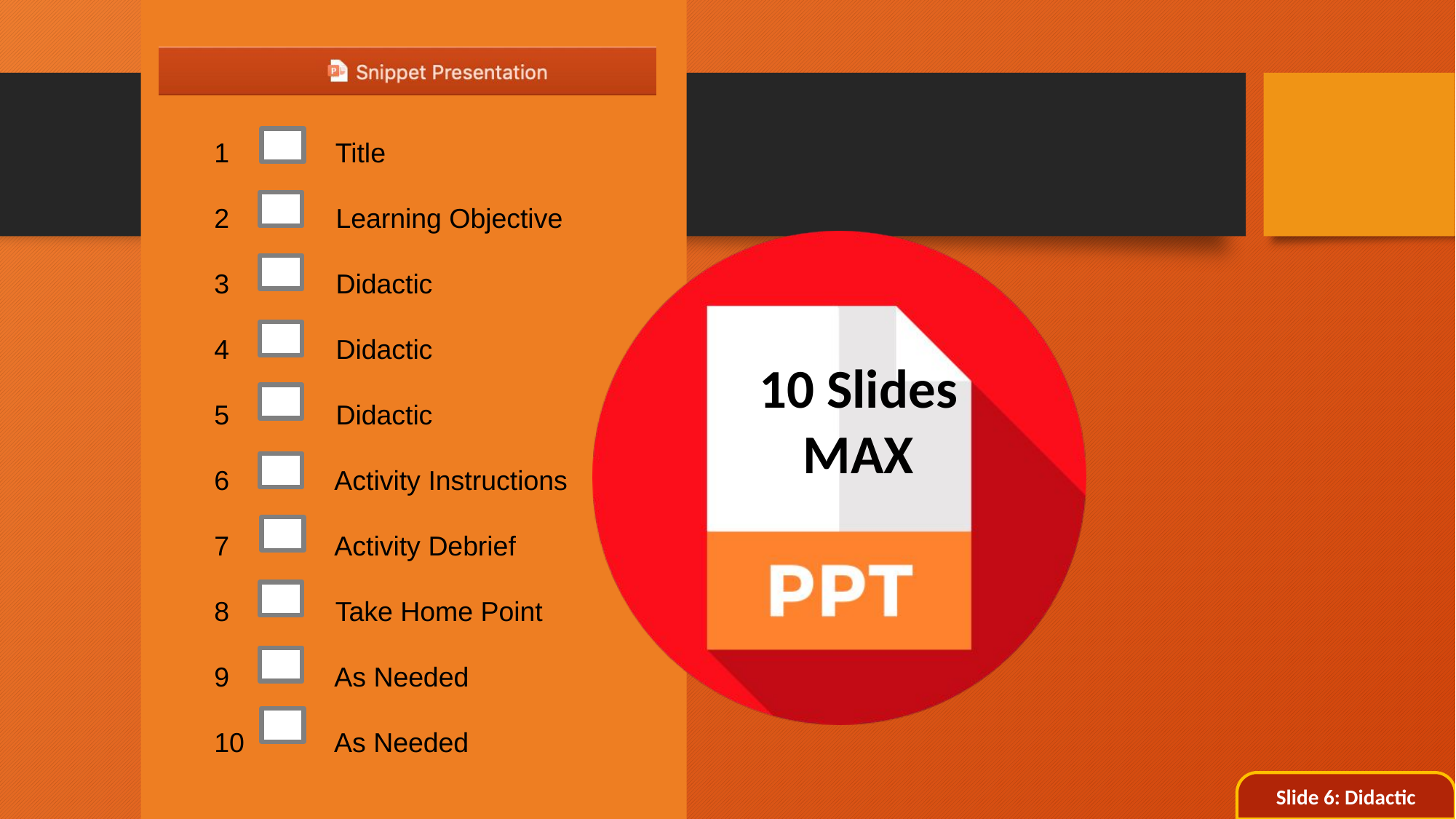

1 Title
2 Learning Objective
3 Didactic
4 Didactic
5 Didactic
6 Activity Instructions
7 Activity Debrief
8 Take Home Point
9 As Needed
10 As Needed
10 Slides MAX
Slide 6: Didactic

## Slide 11
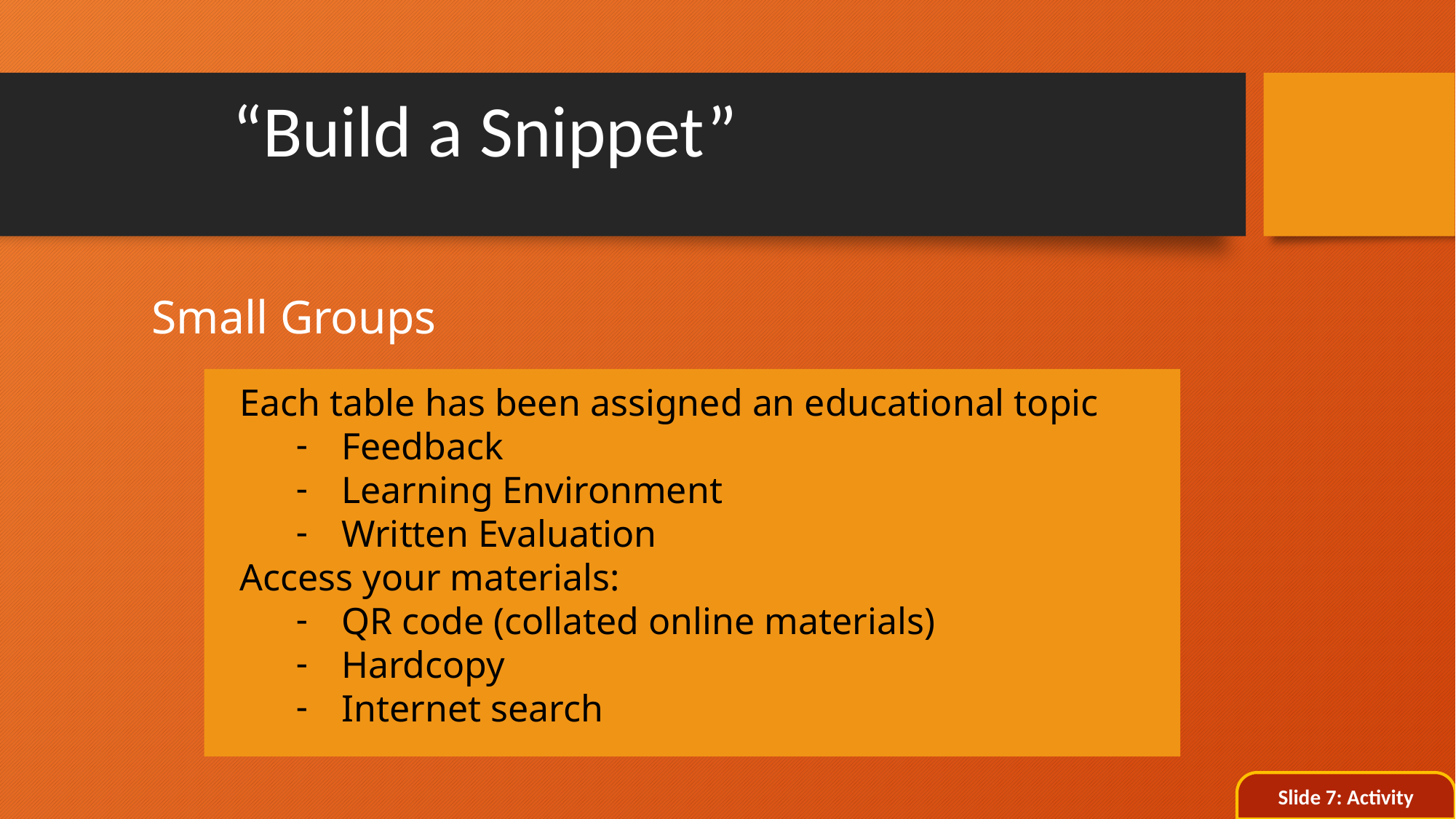

“Build a Snippet”
Small Groups
Each table has been assigned an educational topic
Feedback
Learning Environment
Written Evaluation
Access your materials:
QR code (collated online materials)
Hardcopy
Internet search
Slide 7: Activity

## Slide 12
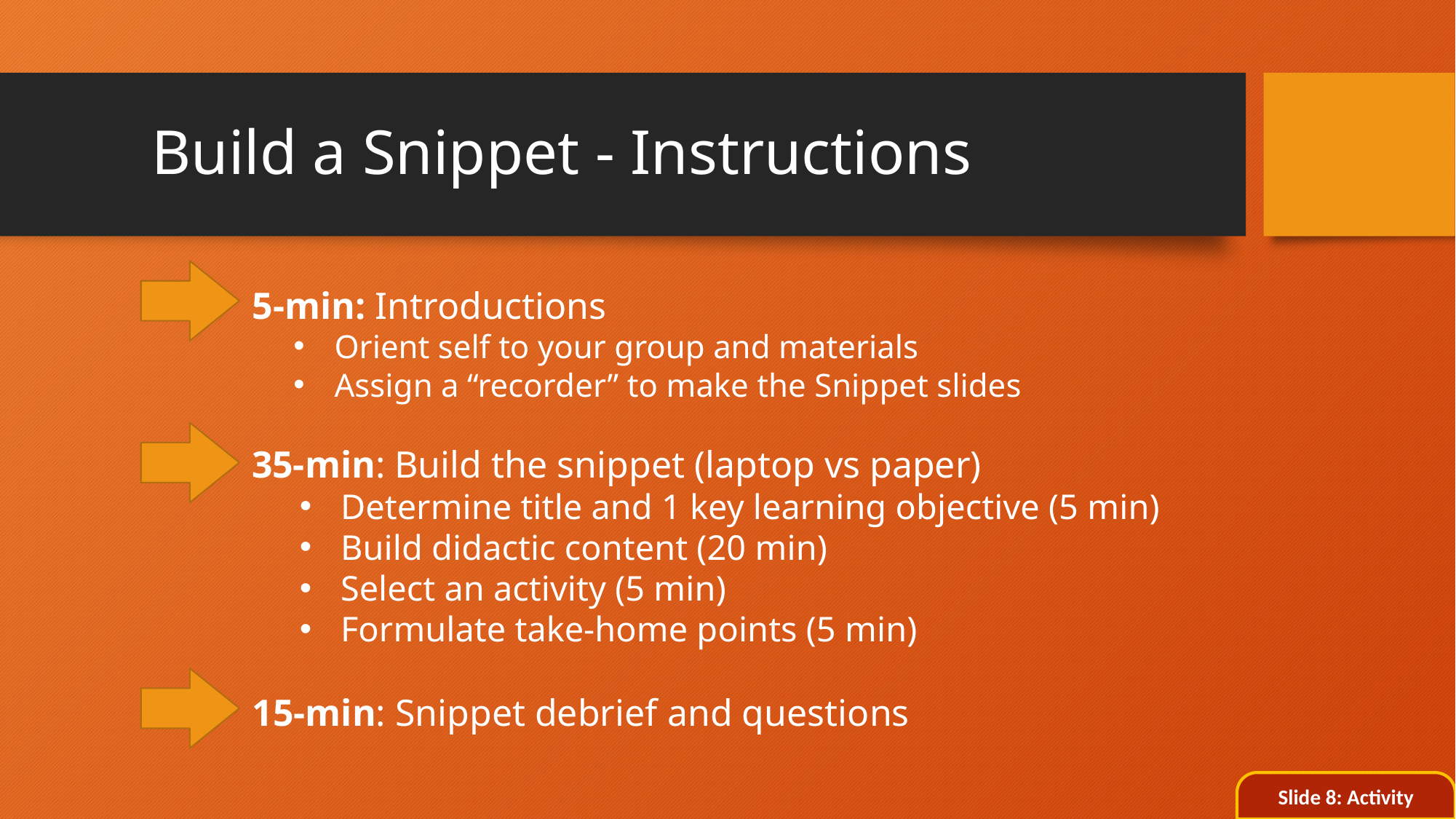

# Build a Snippet - Instructions
5-min: Introductions
Orient self to your group and materials
Assign a “recorder” to make the Snippet slides
35-min: Build the snippet (laptop vs paper)
Determine title and 1 key learning objective (5 min)
Build didactic content (20 min)
Select an activity (5 min)
Formulate take-home points (5 min)
15-min: Snippet debrief and questions
Slide 8: Activity

## Slide 13
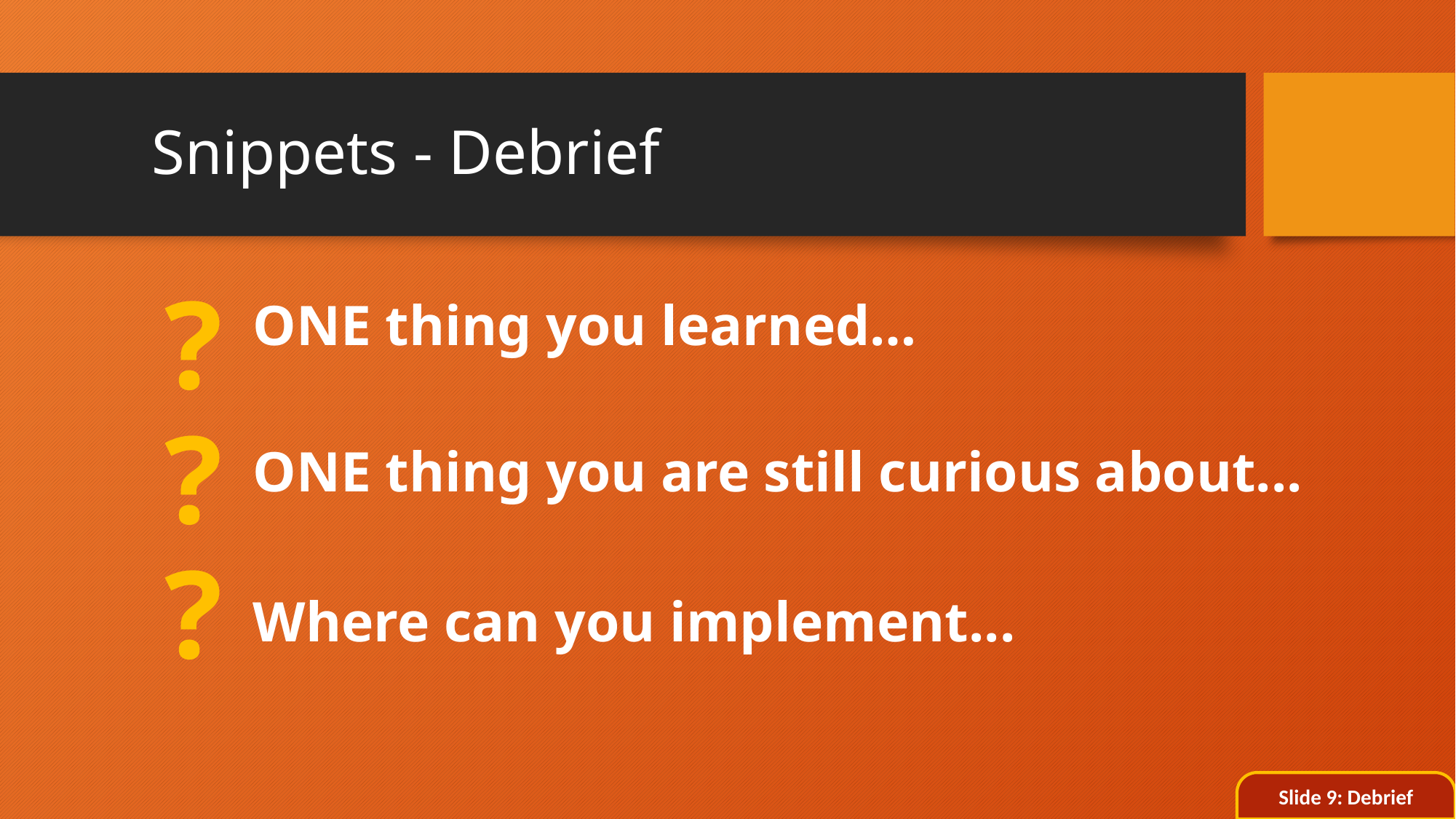

Snippets - Debrief
# ONE thing you learned…
?
?
ONE thing you are still curious about...
?
Where can you implement...
Slide 9: Debrief

## Slide 14
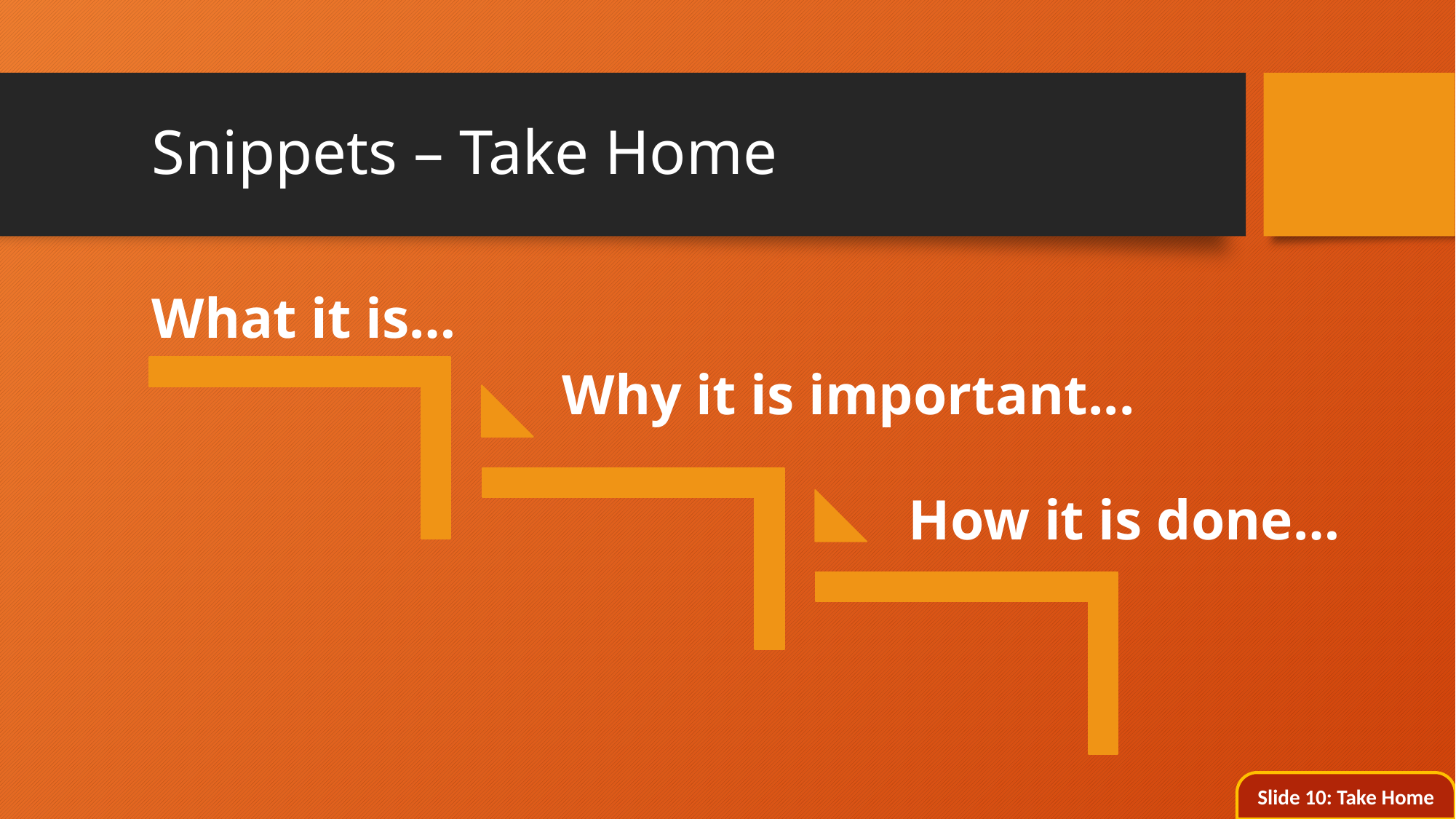

Snippets – Take Home
What it is…
Why it is important...
How it is done…
Slide 10: Take Home

## Slide 15
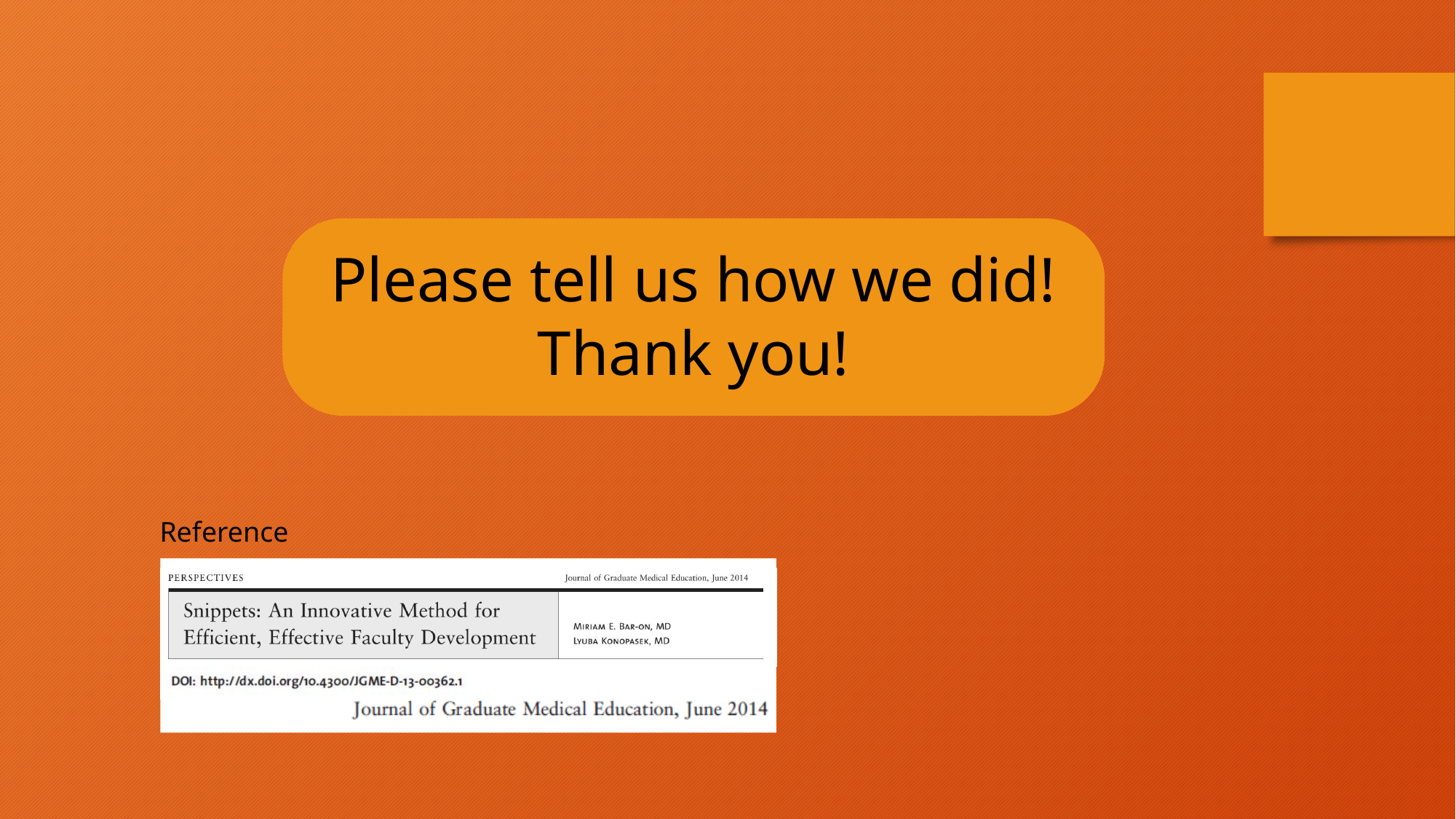

Please tell us how we did!
Thank you!
Reference
