# Supplementary material for: Got 15? Try Faculty Development on the Fly: A Snippets Workshop for Microlearning
Source: MedEdPORTAL. 2021 Jun 14;17:11161. doi: 10.15766/mep_2374-8265.11161 (PMC8200375; doi:10.15766/mep_2374-8265.11161)
Supplement: Supplementary file 1 — Snippet Presentation.pptxSession Plan.docxParticipant Email Message.docxSnippet Template.pptxCurated Materials Learning Environment.docxSmall-Group Instructions.docxExample of Completed Snippet.pptxWorkshop Evaluation.docx [file mep_2374-8265.11161-s001.zip › C. Participant Email Message.docx]

Greetings!

Thank you for registering for the **Faculty Development on the Fly** workshop which will occur on [insert date] from [insert time] in the [insert location].

We are excited to share with you this innovative and proven approach to faculty development through snippets. To optimize your learning and leave with something you can use, this will be a **working** session.

**Please plan to bring:**

1. Your laptop/tablet – something you can use to access the internet, create materials, and upload to a shared file.
2. An interest in creating a brief faculty development session covering one of the following topics:
   - [insert topic name and table number]
   - [add each topic and table number as separate bullets]

When you arrive find the table number that coincides with the topic and sit there. You do NOT need to have prior experience in these topics, all expertise levels are welcome.

1. A willingness to experiment and share your perspective.

We look forward to seeing you!

[insert names of facilitators]
