# Supplementary material for: Got 15? Try Faculty Development on the Fly: A Snippets Workshop for Microlearning
Source: MedEdPORTAL. 2021 Jun 14;17:11161. doi: 10.15766/mep_2374-8265.11161 (PMC8200375; doi:10.15766/mep_2374-8265.11161)
Supplement: Supplementary file 1 — Snippet Presentation.pptxSession Plan.docxParticipant Email Message.docxSnippet Template.pptxCurated Materials Learning Environment.docxSmall-Group Instructions.docxExample of Completed Snippet.pptxWorkshop Evaluation.docx [file mep_2374-8265.11161-s001.zip › D. Snippet Template.pptx]

## Slide 1
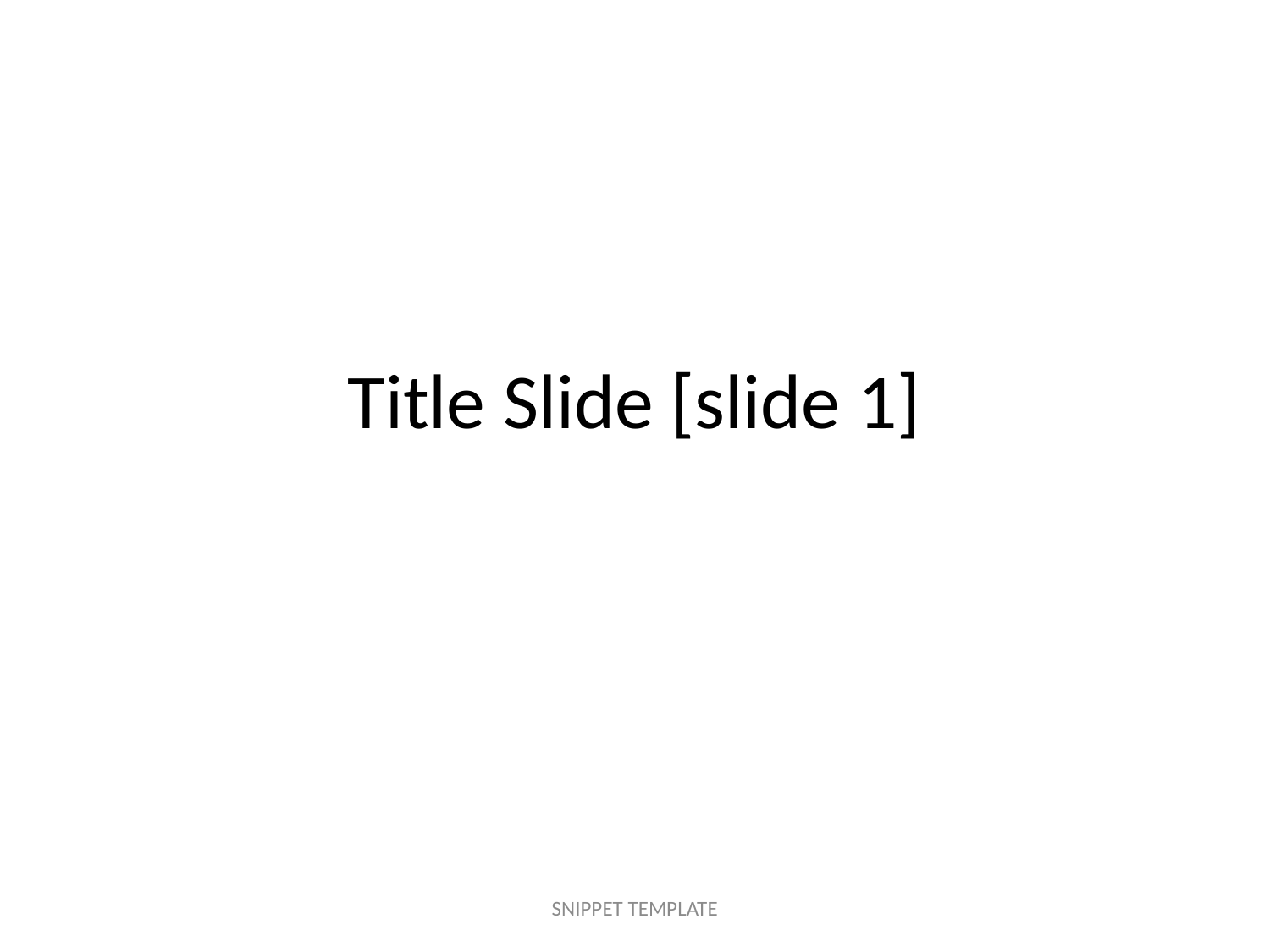

# Title Slide [slide 1]
SNIPPET TEMPLATE

## Slide 2
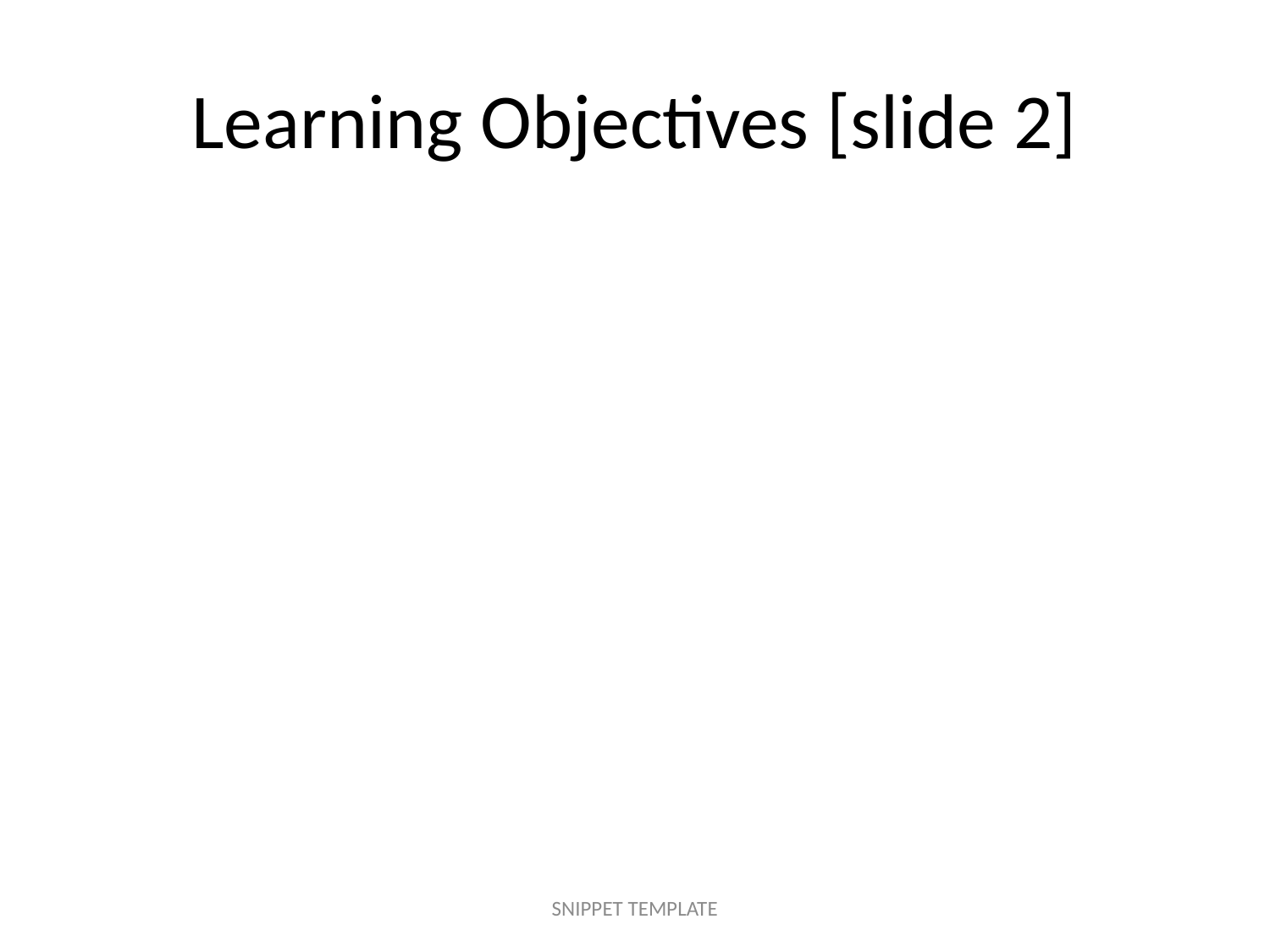

# Learning Objectives [slide 2]
SNIPPET TEMPLATE

## Slide 3
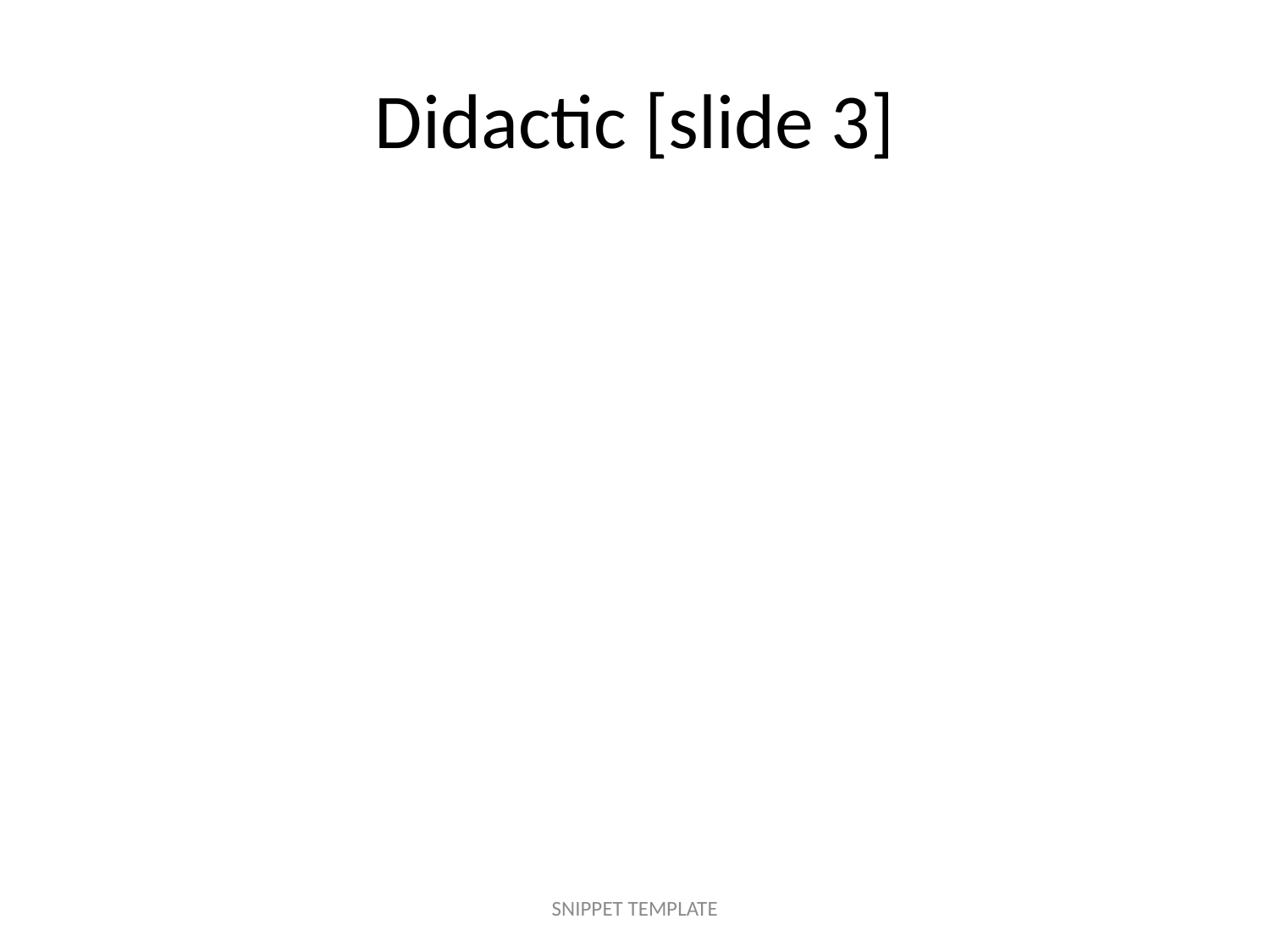

# Didactic [slide 3]
SNIPPET TEMPLATE

## Slide 4
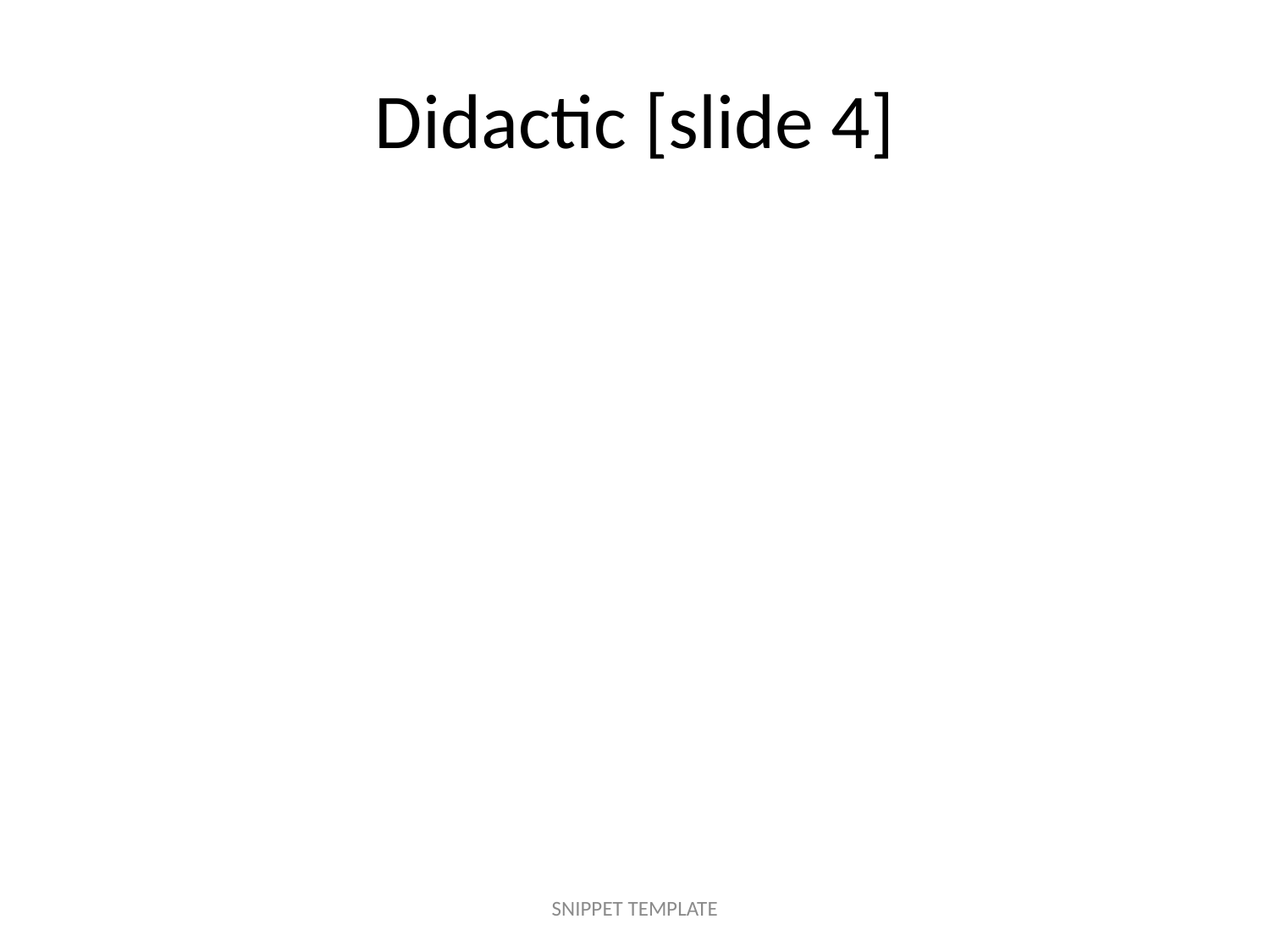

# Didactic [slide 4]
SNIPPET TEMPLATE

## Slide 5
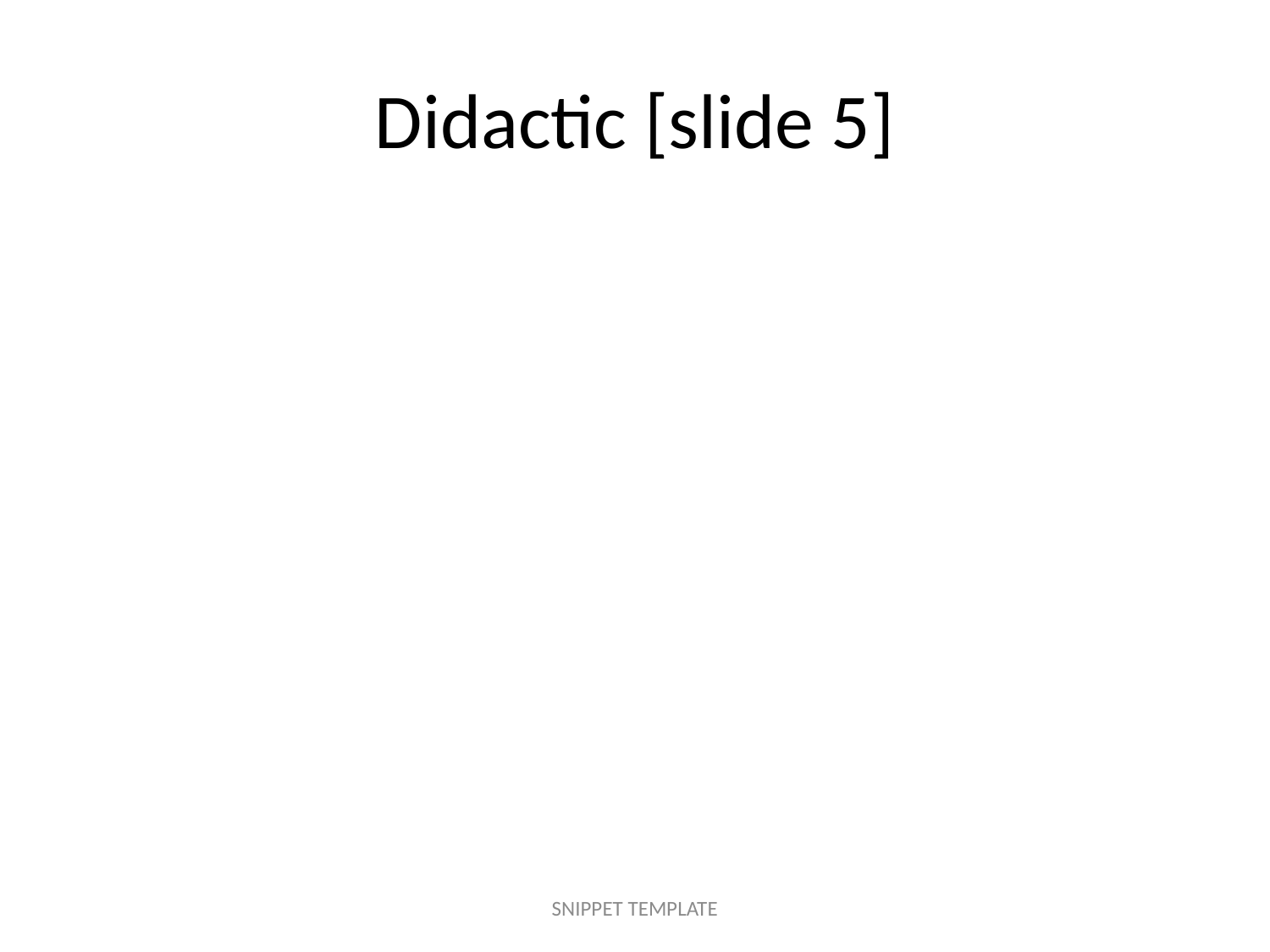

# Didactic [slide 5]
SNIPPET TEMPLATE

## Slide 6
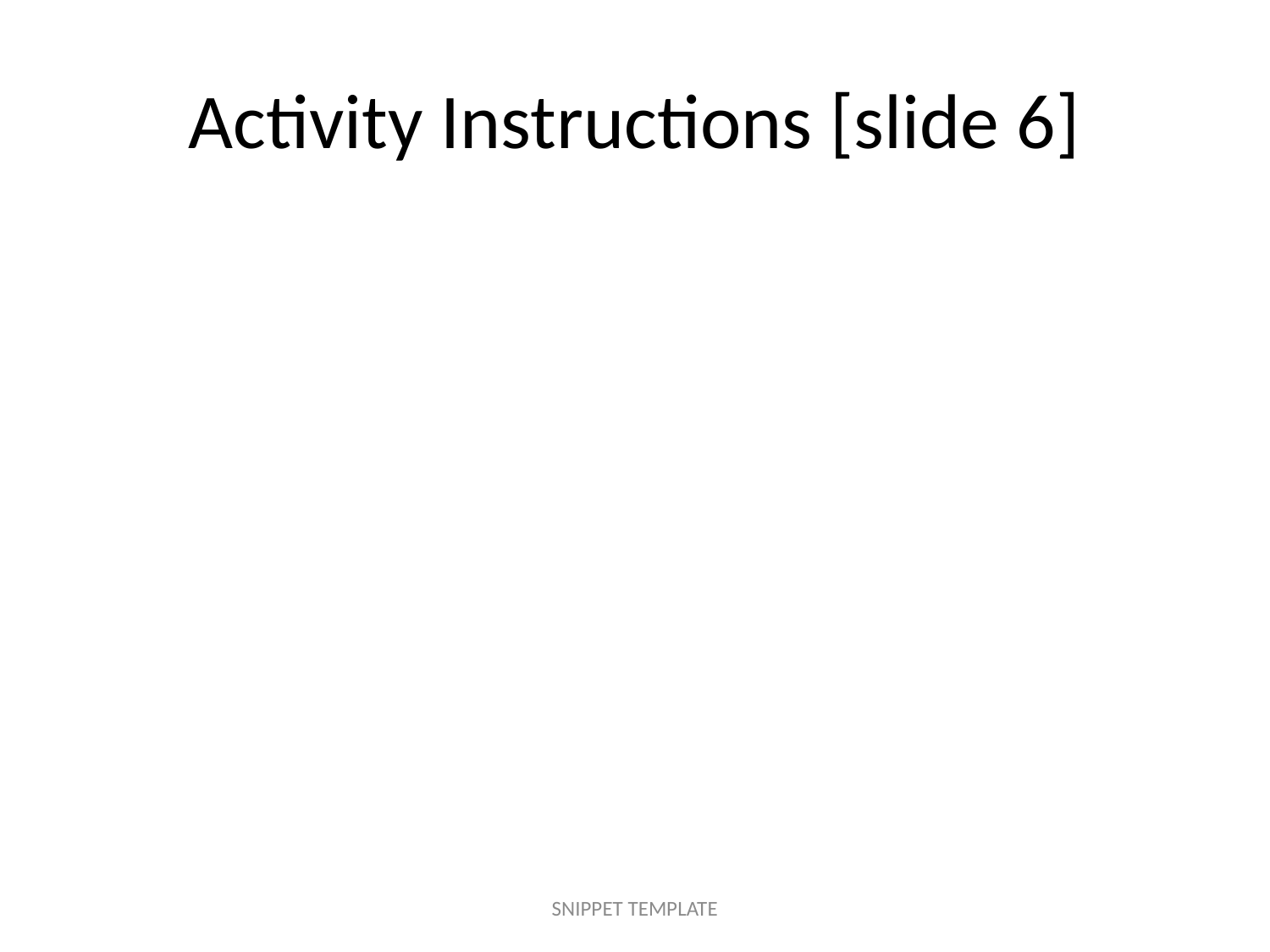

# Activity Instructions [slide 6]
SNIPPET TEMPLATE

## Slide 7
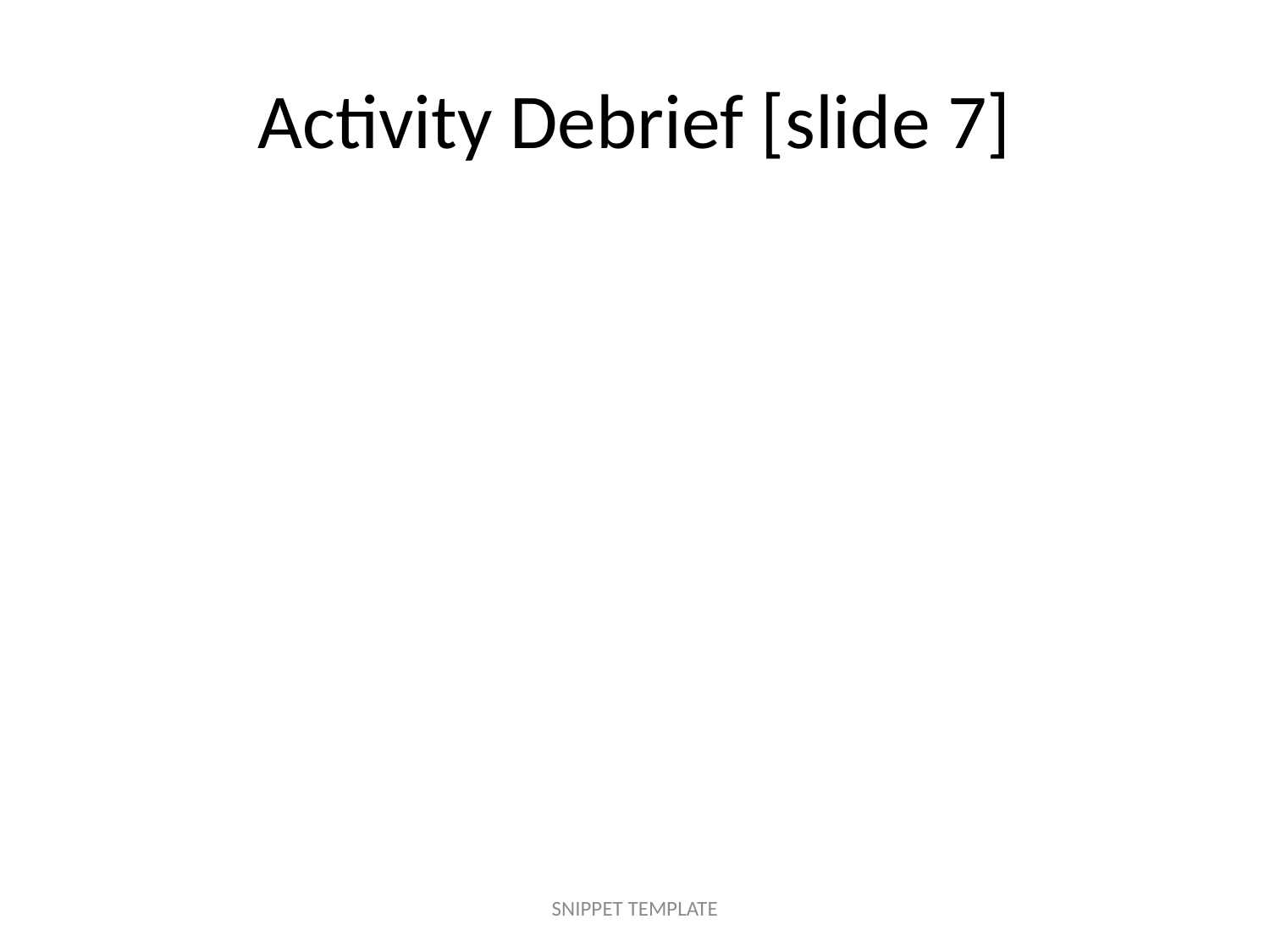

# Activity Debrief [slide 7]
SNIPPET TEMPLATE

## Slide 8
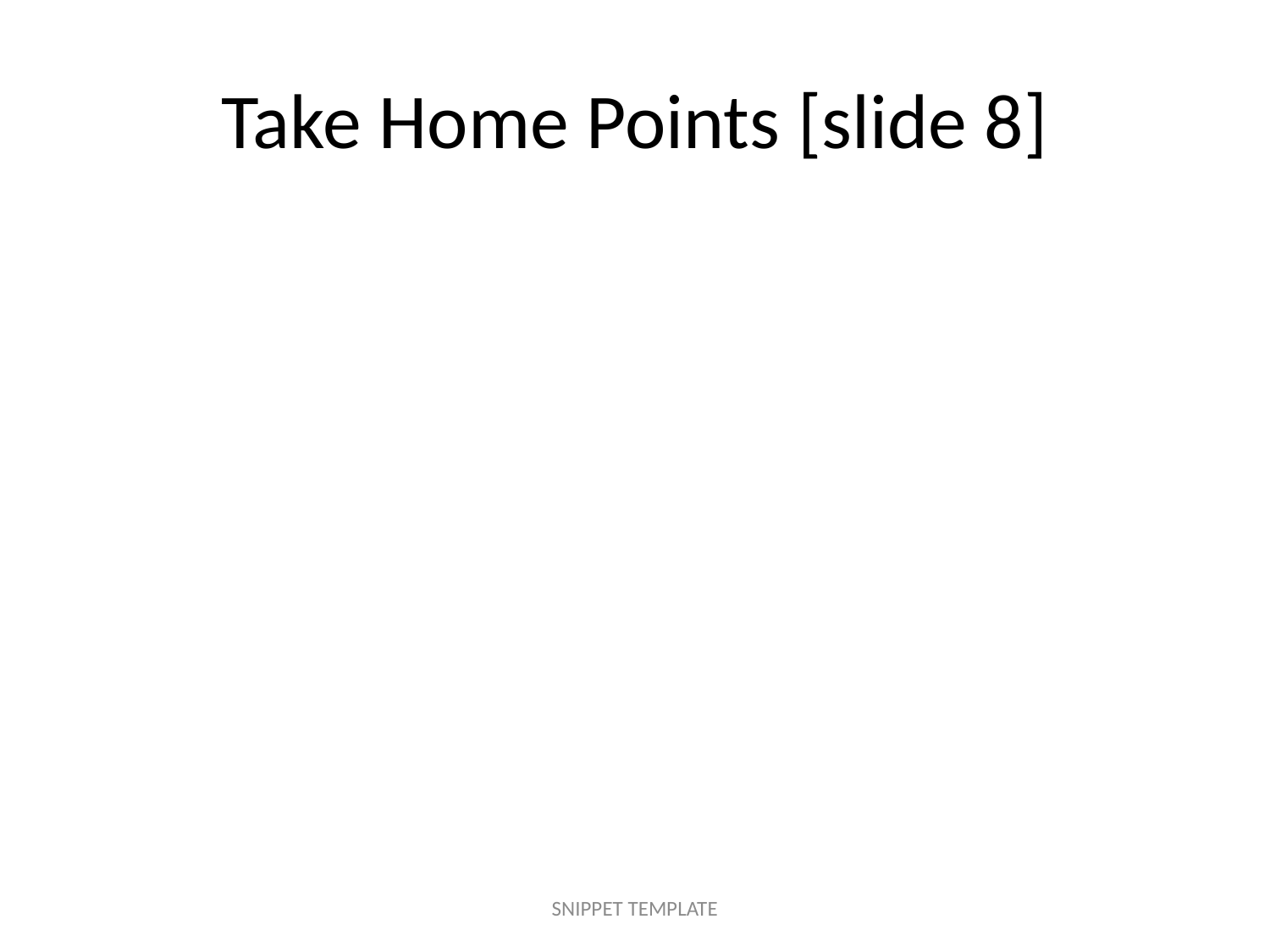

# Take Home Points [slide 8]
SNIPPET TEMPLATE

## Slide 9
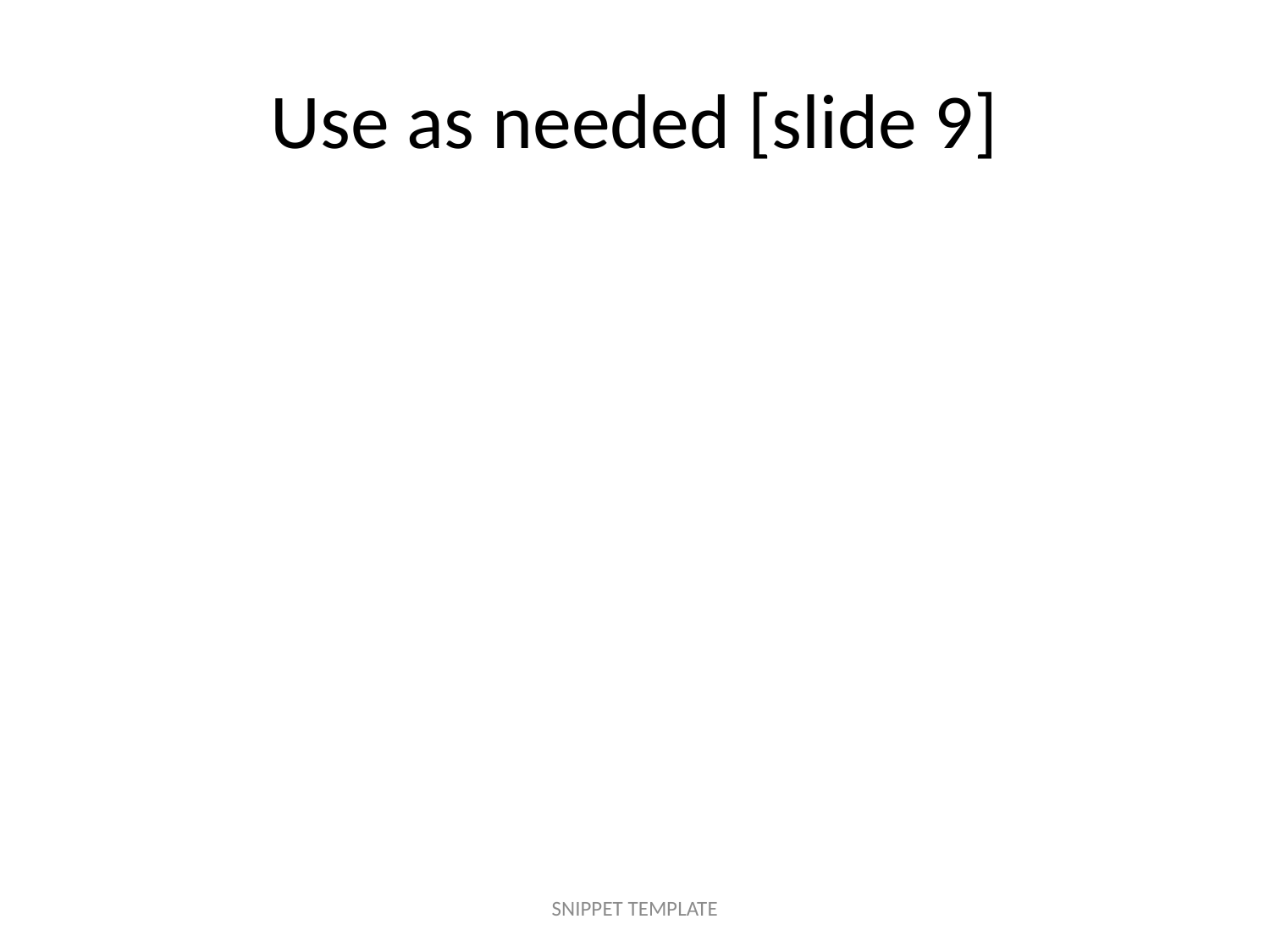

# Use as needed [slide 9]
SNIPPET TEMPLATE

## Slide 10
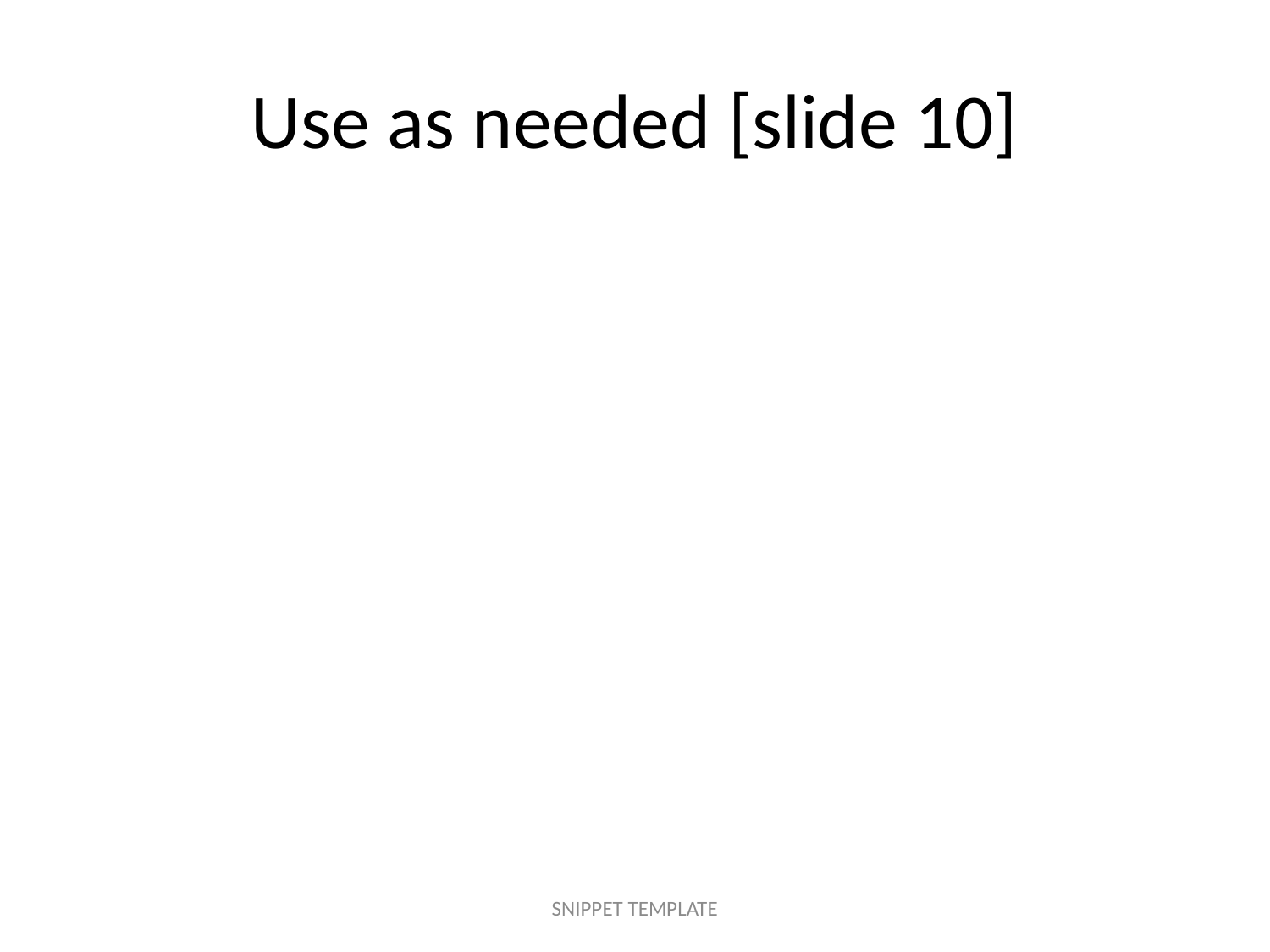

# Use as needed [slide 10]
SNIPPET TEMPLATE
