# Supplementary material for: Got 15? Try Faculty Development on the Fly: A Snippets Workshop for Microlearning
Source: MedEdPORTAL. 2021 Jun 14;17:11161. doi: 10.15766/mep_2374-8265.11161 (PMC8200375; doi:10.15766/mep_2374-8265.11161)
Supplement: Supplementary file 1 — Snippet Presentation.pptxSession Plan.docxParticipant Email Message.docxSnippet Template.pptxCurated Materials Learning Environment.docxSmall-Group Instructions.docxExample of Completed Snippet.pptxWorkshop Evaluation.docx [file mep_2374-8265.11161-s001.zip › E. Curated Materials Learning Environment.docx]

**Curated materials for building a Faculty Development Snippet related to
Creating a Positive Learning Environment**

Definition of Learning Environment:

The **physical space** (building, desks, offices, communal spaces, lighting, etc.), **social** and **psychologica**l context in which trainees are immersed. Consider the **climate** (safety, inclusion, supportive, collaborative, empathetic, etc.).

**IMPORTANT QUESTIONS TO CONSIDER:**

- What is the role of the learning environment in medical education?
- What are components of an effective learning environment?
- How do you know if a learning environment if positive or negative?
- What strategies promote and facilitate and effective learning environment?

1. **ROLE:**
   - Can be a positive influence, promoting engagement
   - Impacts identify formation (professional and moral development)
   - Impacts amount learned, provides opportunities for application and refinement of knowledge
2. **COMPONENTS**

# Learner

- - Self-directed and self-regulated learning (Self-Determination Theory)
  - Engagement, motivation, choice
  - Learning styles
  - Safety

# Educator

- - Roles:
    - Information provider
    - Role model
    - Facilitator
    - Assessor
    - Curriculum and course planner
    - Resource material creator

From AMEE Guide no. 34: Teaching in the clinical environment. S Ramani, S. Leinster. 2008; 30:347-364

- - Role modeling
    - Quote: “Example is not the main thing in influencing others, it is the only thing.” Albert Schweitzer.
    - Article: Role Models and the Learning Environment: Essential Elements in Effective Medical Education. Maudsley, Robert F. MD Academic Medicine: May 2001 - Volume 76 - Issue 5 - p 432–434
  - Psychological Size and Distance
    - Psychological size is “perceived status one person has relative to another”
    - Psychological distance is the “degree of positive and negative emotional connectedness in a relationship”

Vaugh LM, Baker RC. Psychological size and distance: emphasizing the interpersonal relationship as a pathway to optimal teaching and learning conditions. Medical Education 389100 October 2004. 1053- 1060.

- - - - Impacted by roles, titles, status, reputation AND
      - Use of
        - Criticism/sarcasm
        - Ridicule/humor
        - Terminal statements (never/always, all)
        - Manner of deliver
        - Punishing remarks
        - Complicated language, jargon
        - Failure to use names
        - Overemphasizing specific components (grades, board scores, memberships)

1. **STRATEGIES**
   - Promote a sense of **relatedness** – learner as part of a community
     - Importance of relationships and interactions with faculty, supervisors, peers
   - Promote a sense of **autonomy** - learner ability to initiate learning activities on own (with supervision!)
     - Delegation
     - Clear expectations
   - Promote a sense of **competence/mastery**
     - Clear expectations – aligning expectations with learner’s abilities
     - Constructive feedback focused on growth
     - Escalating challenges
   - Demonstrate **enthusiasm**
   - Be a **Team Player**
   - Show **Empathy**
     - Take an personal interest in learners’ lives
   - Model **Humility**
     - Share experiences and lessons learned from your mistakes
   - Balance **Challenge and Support**
     - clear expectations
     - be approachable for questions, concerns,
     - use growth mindset for feedback (no punitive language)

VIDEOS – SMALL GROUP DISCUSSION

A great collection of videos can be found on here: Rich A, Ackerman S, Patel C, Feldman N, Adams D, Lewis J. Creating a positive learning environment: educational film and discussion guide. MedEdPORTAL Publications. 2015;11:10131. <http://doi.org/10.15766/mep_2374-8265.10131>

**SCENARIO 1: GYNECOLOGY OUTPATIENT CLINIC *(90 SECONDS)***

# Video Link: (to be added by Facilitators)

Question to consider:

- How would you have acted differently if you were the faculty member? If you were the student?
  - What if you witnessed this of a colleague? How might you approach/counsel/coach your colleague or a medical student in this position?

Takeaways:

- Staff should serve as advocates for the learner when soliciting permission from patients.
  - Benign Neglect: Where learners are excluded from meaningful learning experiences (Gan & Snell, 2014)

**SCENARIO 2: QUESTIONING TO ASSESS UNDERSTANDING *(< 3 MINUTES)***

**Video Link**: (to be added by Facilitators)

Questions to consider:

- Did this scenario demonstrate a positive learning environment…or a negative one?
- Did you notice that the attending referred to the male student by his last name and the female student by her first name?

Takeaways:

- Notice how the behavior of the faculty member affects the subsequent behavior of the Sr. Resident
- Learners who experience a negative learning environment are more likely to perpetuate it down the line.
- Unconscious changes in how we refer to learners can affect their sense of belonging.

**SCENARIO 3: DISENGAGED STAFF *(90 SECONDS)***

# Video Link: (to be added by Facilitators)

Question to consider:

- Have you ever had an experience like this?
  - How would you counsel the learner? How would you counsel the faculty member?

Takeaways:

- When learners reach out - stop, focus and listen to them.
- Use non-patient time to discuss medical knowledge, and patient care practices.
- Get to know your learners.
- Media multitasking has been associated with negative psychosocial and cognitive impacts (Loh & Kanai, 2014)

Adapted from: Rich A, Ackerman S, Patel C, Feldman N, Adams D, Lewis J. Creating a positive learning environment: educational film and discussion guide. MedEdPORTAL Publications. 2015;11:10131. <http://doi.org/10.15766/mep_2374-8265.10131>

**IMAGES:** Use your preferred search strategy to find images to include for Snippet Build. Here are a few examples**:**

| **Website** | **URL** | **Info** |
| --- | --- | --- |
| 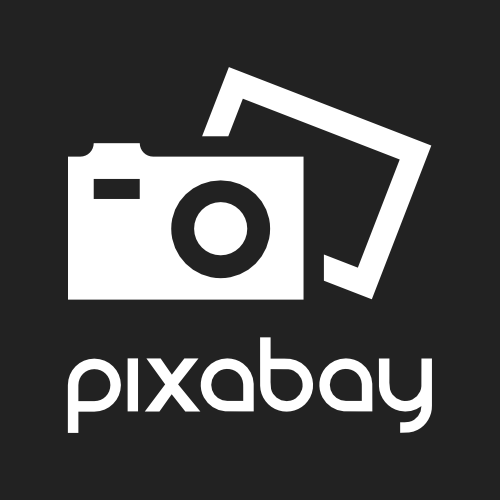 | https://pixabay.com | Sharing over 15 million royalty free images and videos you can use anywhere. All contents are released under Creative Commons CC0, which makes them safe to use without asking for permission or giving credit to the artist, even for commercial purposes. |
| 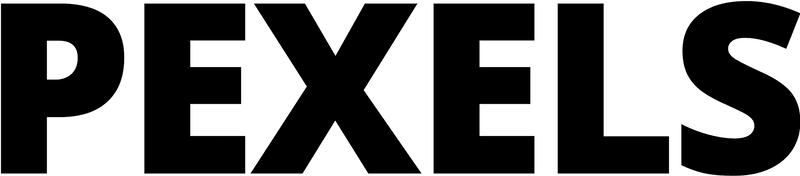 | https://[www.pexels.com](http://www.pexels.com/) | All photos on Pexels are free for personal and commercial purposes |
| 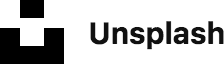 | https://unsplash.com | Over 550.000 free, high- resolution, photos brought to you by the world’s most generous community of photographers.  Beautiful, free photos, to do whatever you want with them. Join unsplash photo-library for free, to gain access to fresh photos that are hand-selected every day. |
| 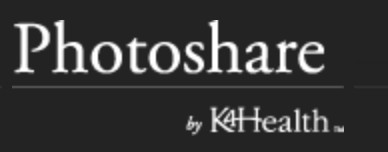 | https://[www.photoshare.org/](http://www.photoshare.org/) | Public health, international health and development images. **Free** for nonprofit and educational use. |
| 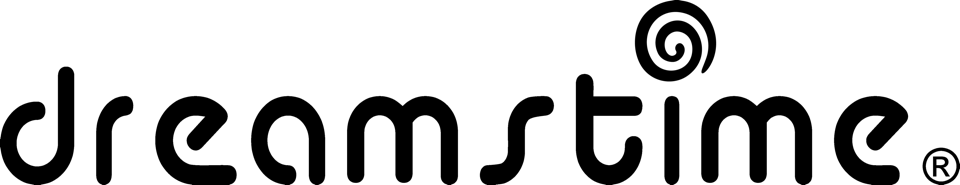 | https://[www.dreamstime.com/free-photos](http://www.dreamstime.com/free-photos) | Royalty-Free stock photography website |
| 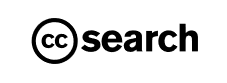 | <https://ccsearch-dev.creativecommons.org/> | All our content is under Creative Commons licenses or in the public domain. |
