# Supplementary material for: Got 15? Try Faculty Development on the Fly: A Snippets Workshop for Microlearning
Source: MedEdPORTAL. 2021 Jun 14;17:11161. doi: 10.15766/mep_2374-8265.11161 (PMC8200375; doi:10.15766/mep_2374-8265.11161)
Supplement: Supplementary file 1 — Snippet Presentation.pptxSession Plan.docxParticipant Email Message.docxSnippet Template.pptxCurated Materials Learning Environment.docxSmall-Group Instructions.docxExample of Completed Snippet.pptxWorkshop Evaluation.docx [file mep_2374-8265.11161-s001.zip › F. Small-Group Instructions.docx]

**Build a Snippet – Small Group Instructions**

**Objective:** At the completion of this activity, each table will have a completed Snippet that could be presented to the large group.

1. Each table has been assigned an educational topic
   *This could be the same topic for all tables or multiple topics*
2. Access your materials
   1. Hardcopy (provided by workshop facilitators_ **Appendix E**)
   2. Internet search (phone or laptop)
   3. QR code (collated online materials)

| **LEARNING ENVIRONMENT**  *This is an* ***example*** *of how we leveraged shared folders within Google Drive and directed participants to access pre-curated materials through a QR code and a hyperlink. We leveraged QR codes and bit.ly hyperlinks.* |
| --- |
| 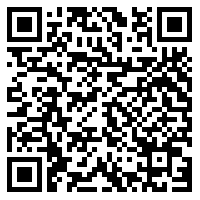 |
| <https://bit.ly/2TDxJmt> |

3. Introductions **(5 minutes)**

a. Orient self to your partners and materials

b. Assign roles

4. Build the snippet (laptop vs paper) **(40 minutes)**

a. Determine title and 1 key learning objective (5 minutes)

b. Build didactic content (20 minutes)

c. Select an activity (10 minutes)

d. Formulate take-home points (5 minutes)

e. Upload your snippet to a shared folder to share!

5. Snippet Debrief & Questions **(15 minutes)***Typically one group would present their Snippet to the large group. Workshop facilitators would moderate a large group discussion and conclude with Q & A.*

**** If your group needs help, raise your hand and a facilitator will assist you****
