# Supplementary material for: Got 15? Try Faculty Development on the Fly: A Snippets Workshop for Microlearning
Source: MedEdPORTAL. 2021 Jun 14;17:11161. doi: 10.15766/mep_2374-8265.11161 (PMC8200375; doi:10.15766/mep_2374-8265.11161)
Supplement: Supplementary file 1 — Snippet Presentation.pptxSession Plan.docxParticipant Email Message.docxSnippet Template.pptxCurated Materials Learning Environment.docxSmall-Group Instructions.docxExample of Completed Snippet.pptxWorkshop Evaluation.docx [file mep_2374-8265.11161-s001.zip › G. Example of Completed Snippet.pptx]

## Slide 1
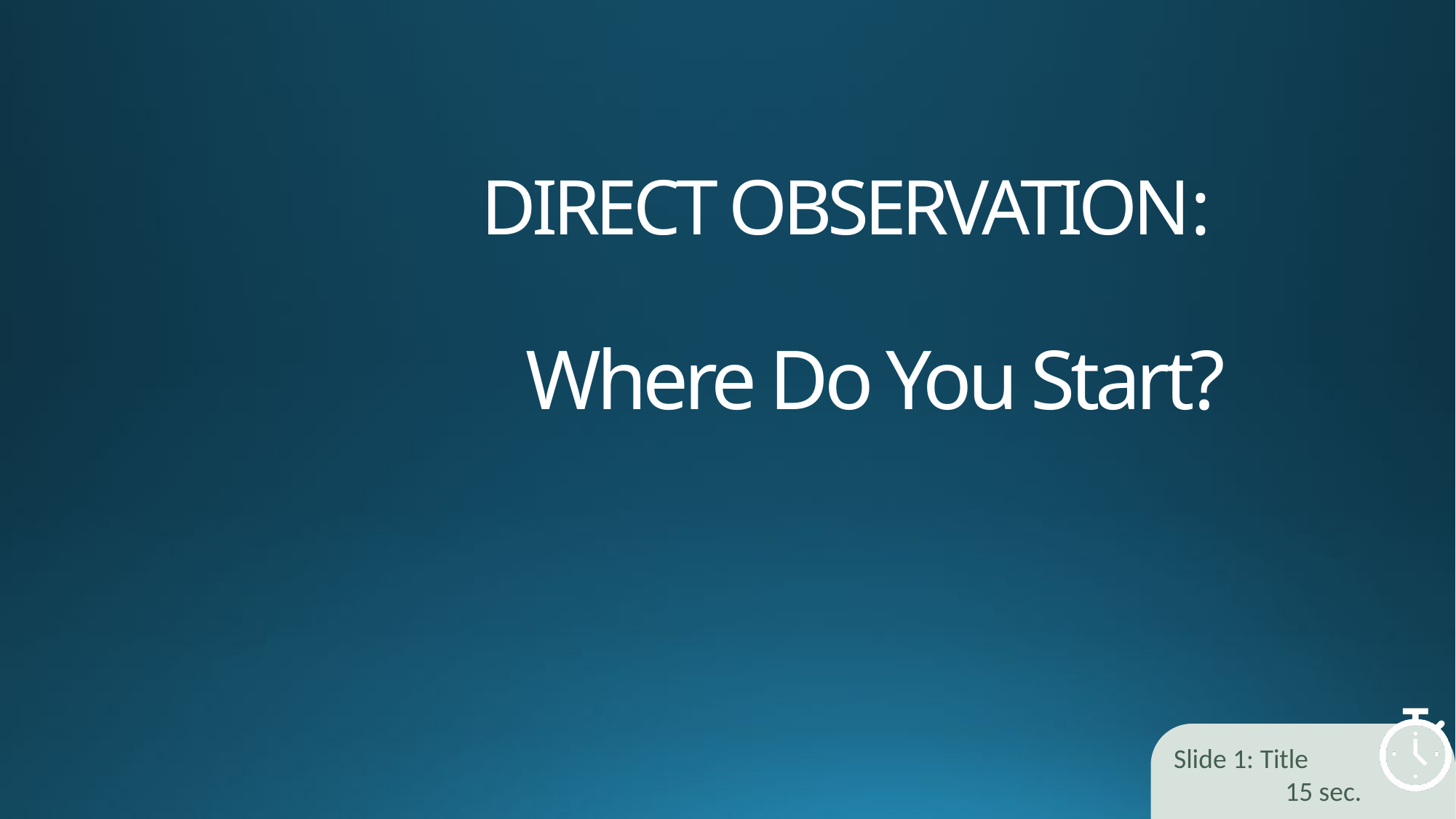

# Direct Observation: Where Do You Start?
Slide 1: Title
 15 sec.

## Slide 2
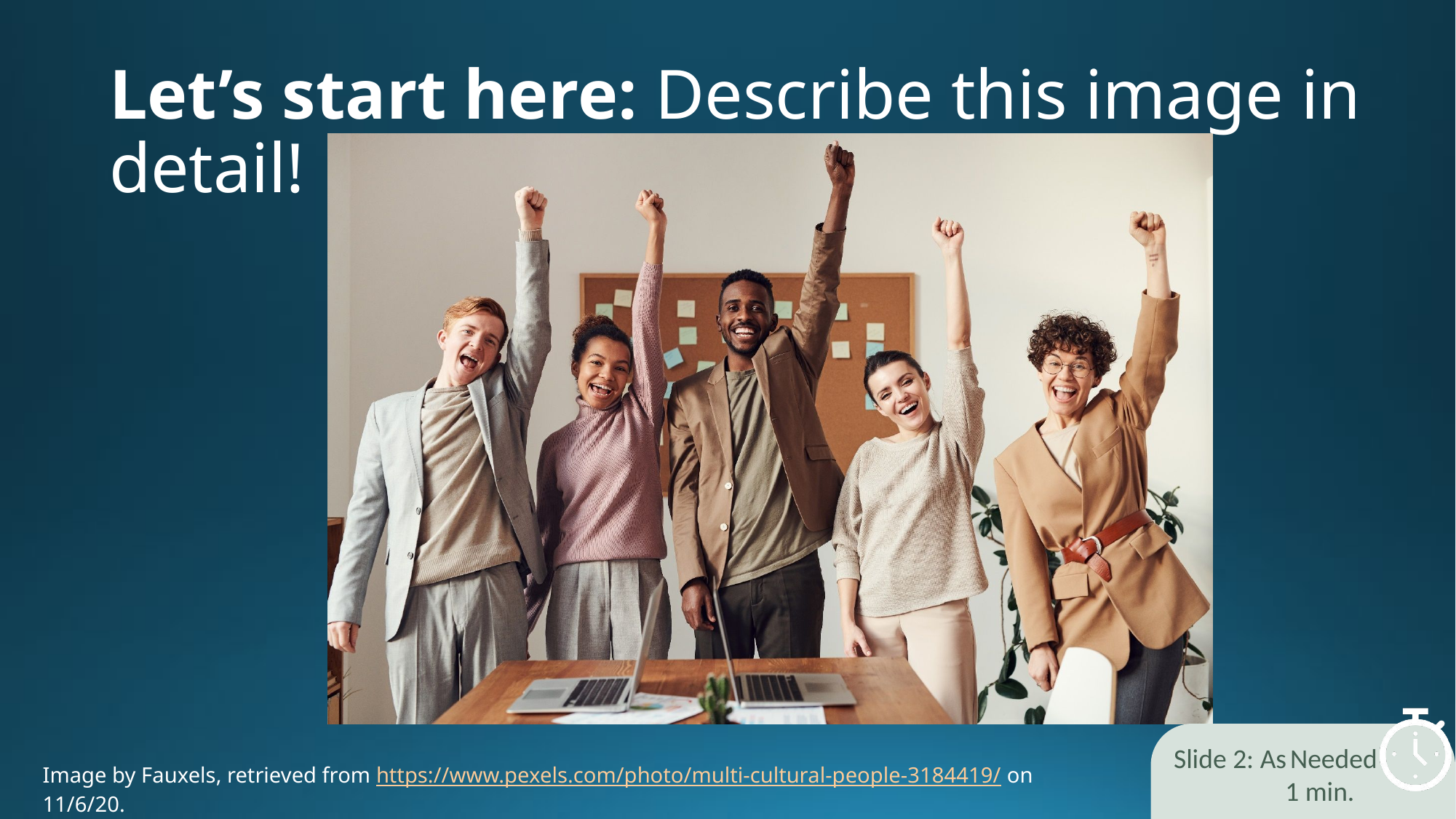

Let’s start here: Describe this image in detail!
Slide 2: As Needed
 1 min.
Image by Fauxels, retrieved from https://www.pexels.com/photo/multi-cultural-people-3184419/ on 11/6/20.
Creative Commons License: public domain.

## Slide 3
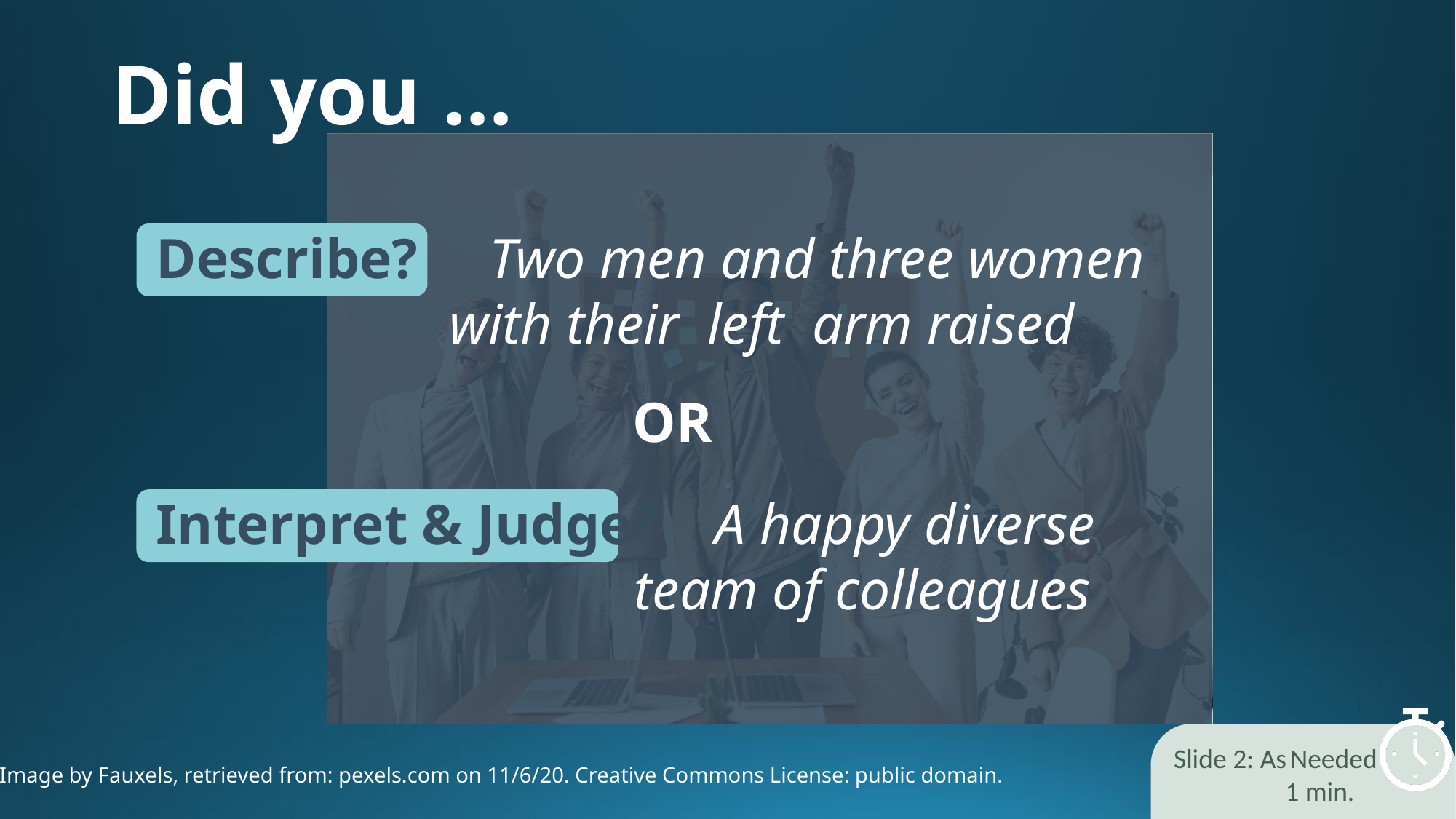

Did you …
Describe? Two men and three women with their left arm raised
OR
Interpret & Judge? A happy diverse team of colleagues
Slide 2: As Needed
 1 min.
Image by Fauxels, retrieved from: pexels.com on 11/6/20. Creative Commons License: public domain.

## Slide 4
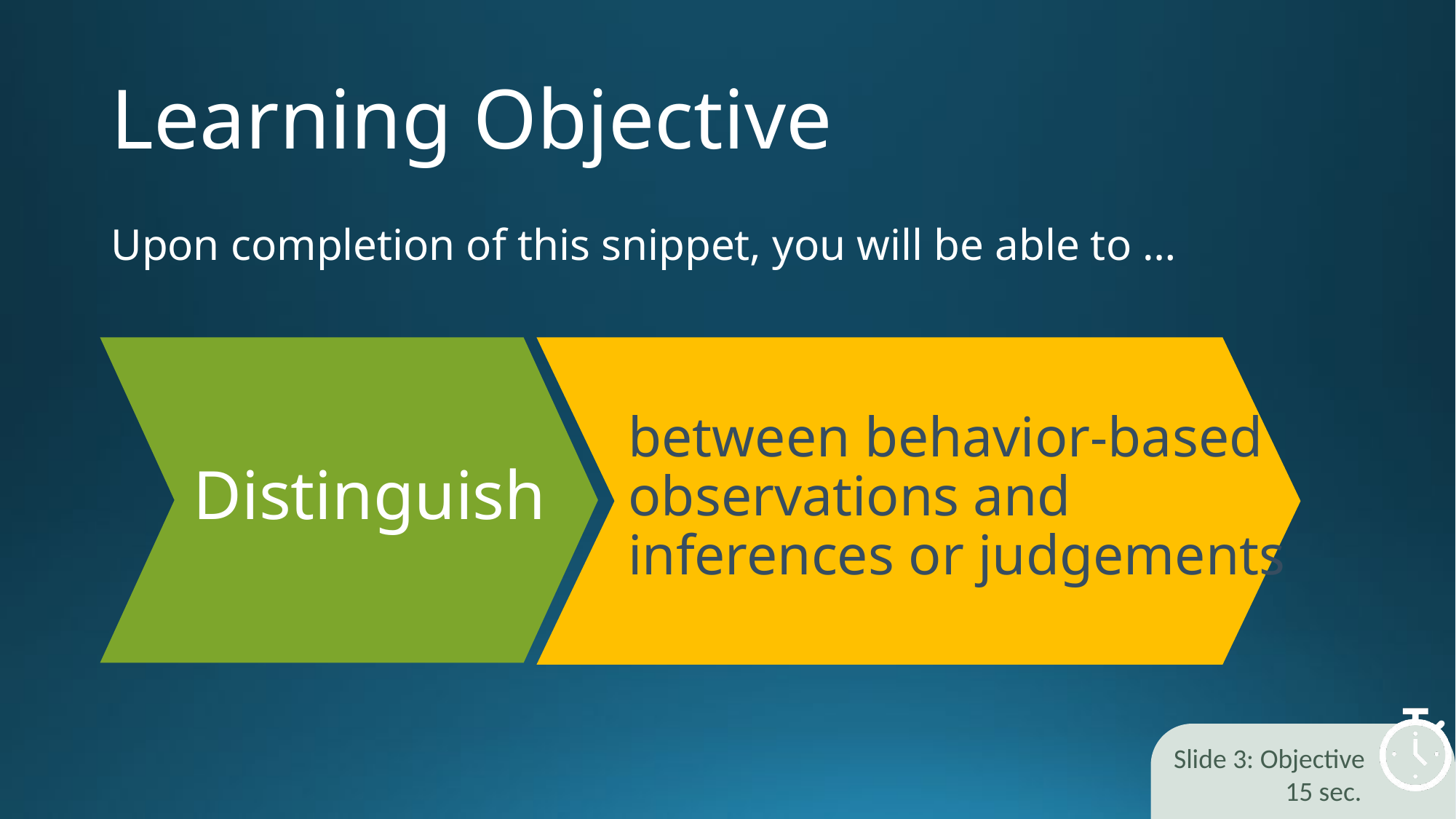

# Learning Objective
Upon completion of this snippet, you will be able to …
between behavior-based observations and
inferences or judgements
Distinguish
Slide 3: Objective
 15 sec.

## Slide 5
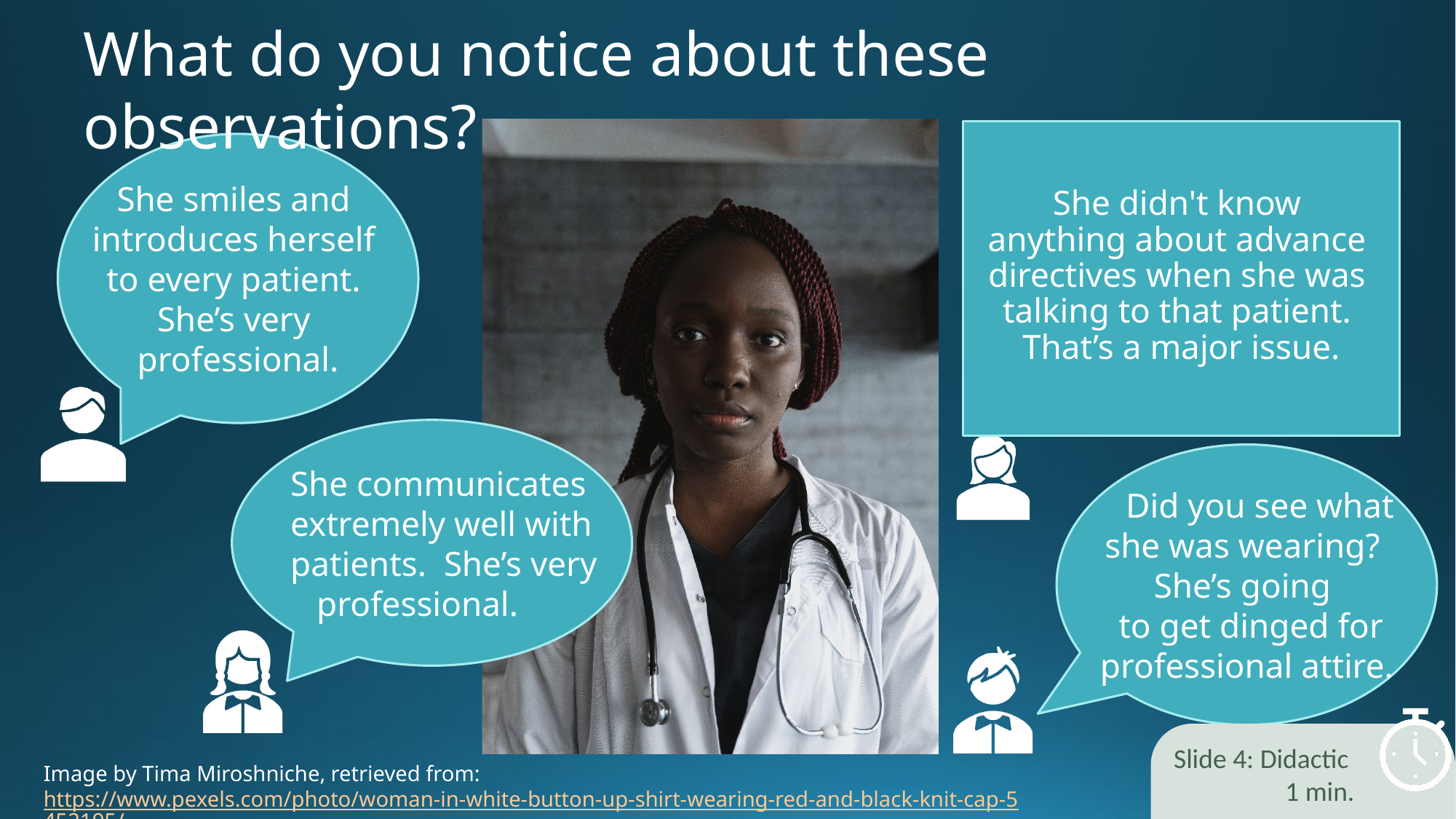

What do you notice about these observations?
She didn't know
anything about advance
directives when she was
talking to that patient.
That’s a major issue.
She smiles and
introduces herself
to every patient.
She’s very
professional.
She communicates
extremely well with
patients. She’s very
 professional.
 Did you see what
she was wearing?
She’s going
 to get dinged for
professional attire.
Slide 4: Didactic
 1 min.
Image by Tima Miroshniche, retrieved from: https://www.pexels.com/photo/woman-in-white-button-up-shirt-wearing-red-and-black-knit-cap-5452195/ on 11/6/20. Creative Commons License: public domain.

## Slide 6
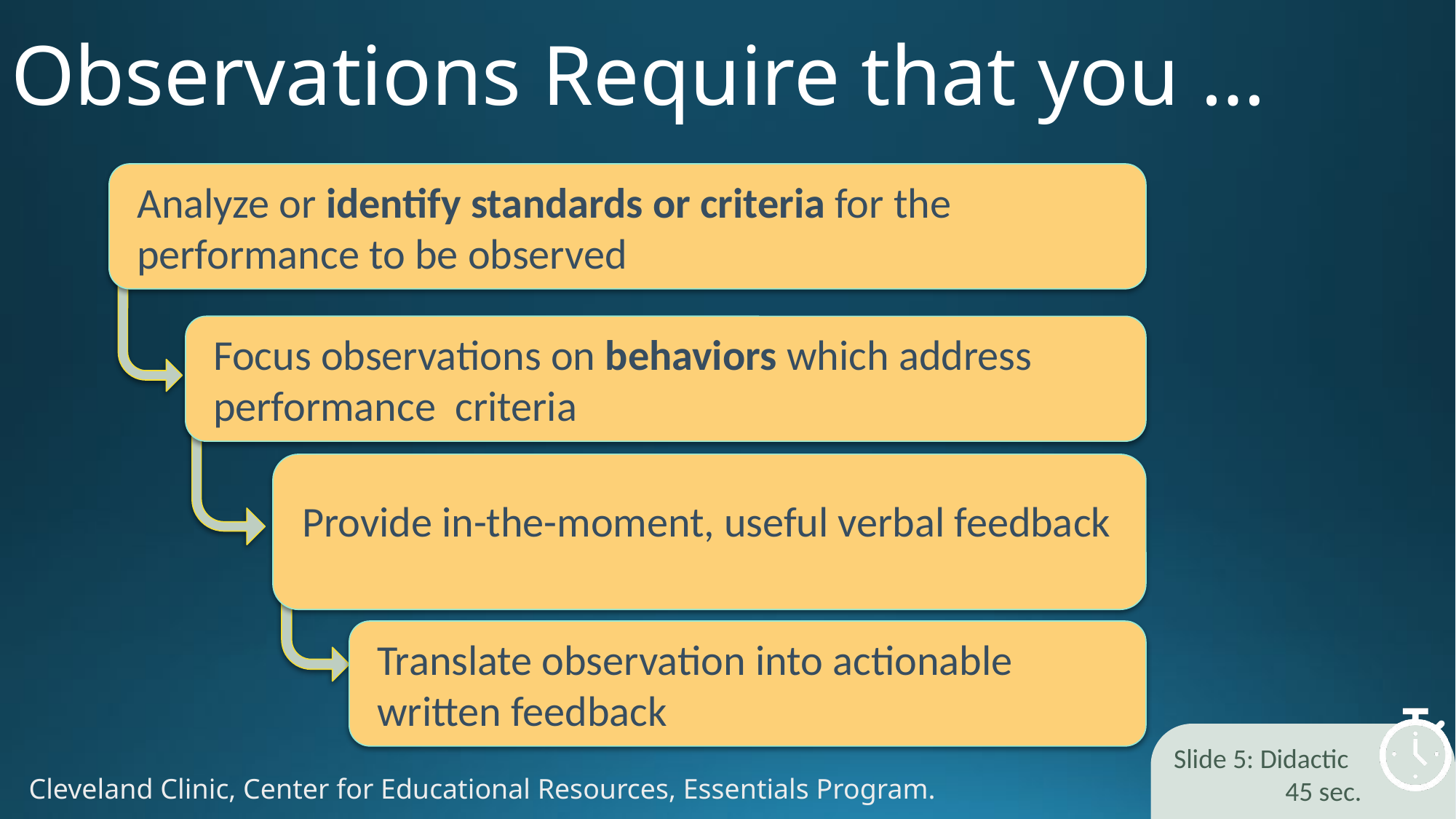

Observations Require that you …
Analyze or identify standards or criteria for the performance to be observed
Focus observations on behaviors which address performance criteria
Provide in-the-moment, useful verbal feedback
Translate observation into actionable written feedback
Slide 5: Didactic
 45 sec.
Cleveland Clinic, Center for Educational Resources, Essentials Program.

## Slide 7
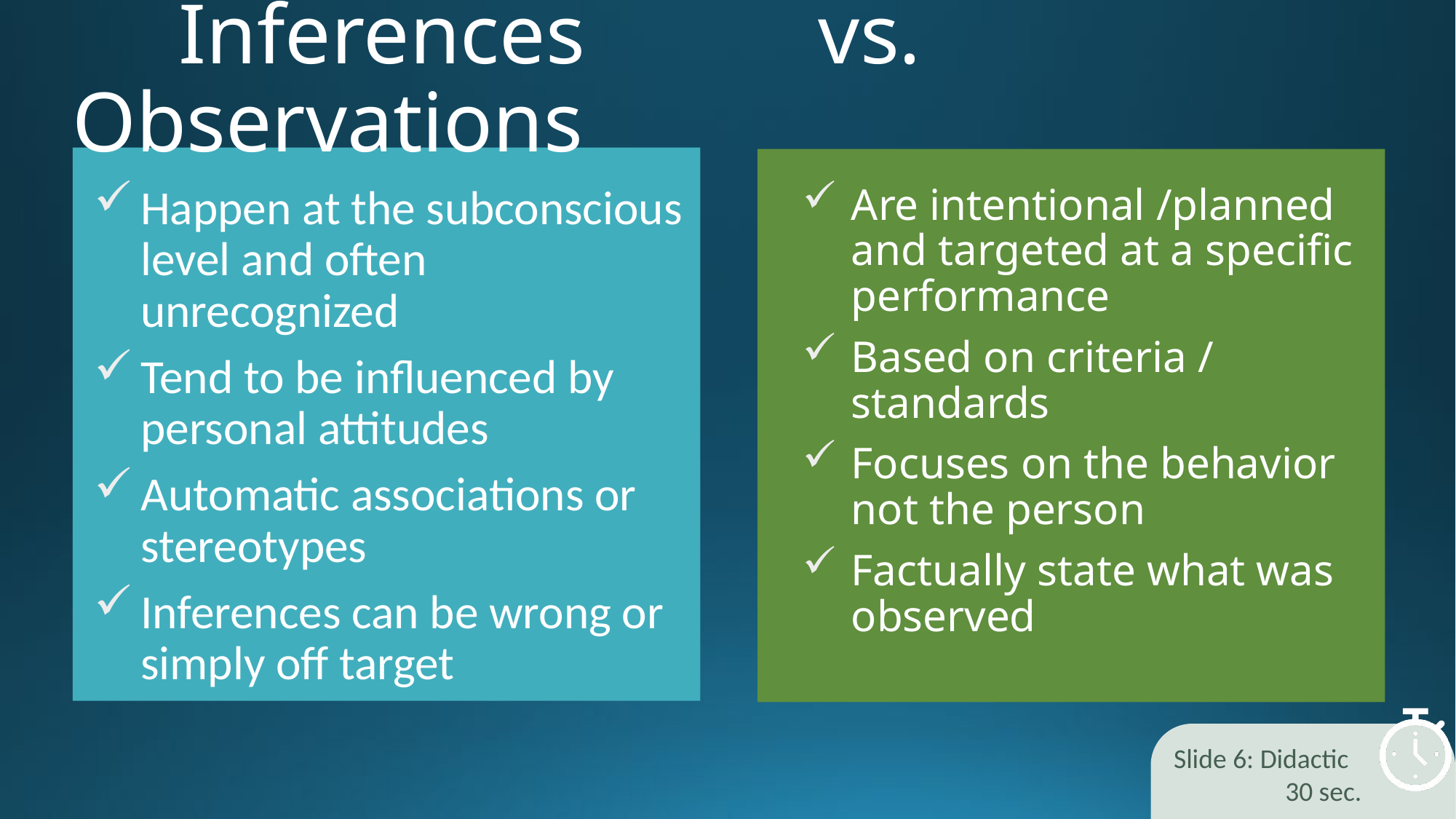

# Inferences vs. Observations
Happen at the subconscious level and often unrecognized
Tend to be influenced by personal attitudes
Automatic associations or stereotypes
Inferences can be wrong or simply off target
Are intentional /planned and targeted at a specific performance
Based on criteria / standards
Focuses on the behavior not the person
Factually state what was observed
Slide 6: Didactic
 30 sec.

## Slide 8
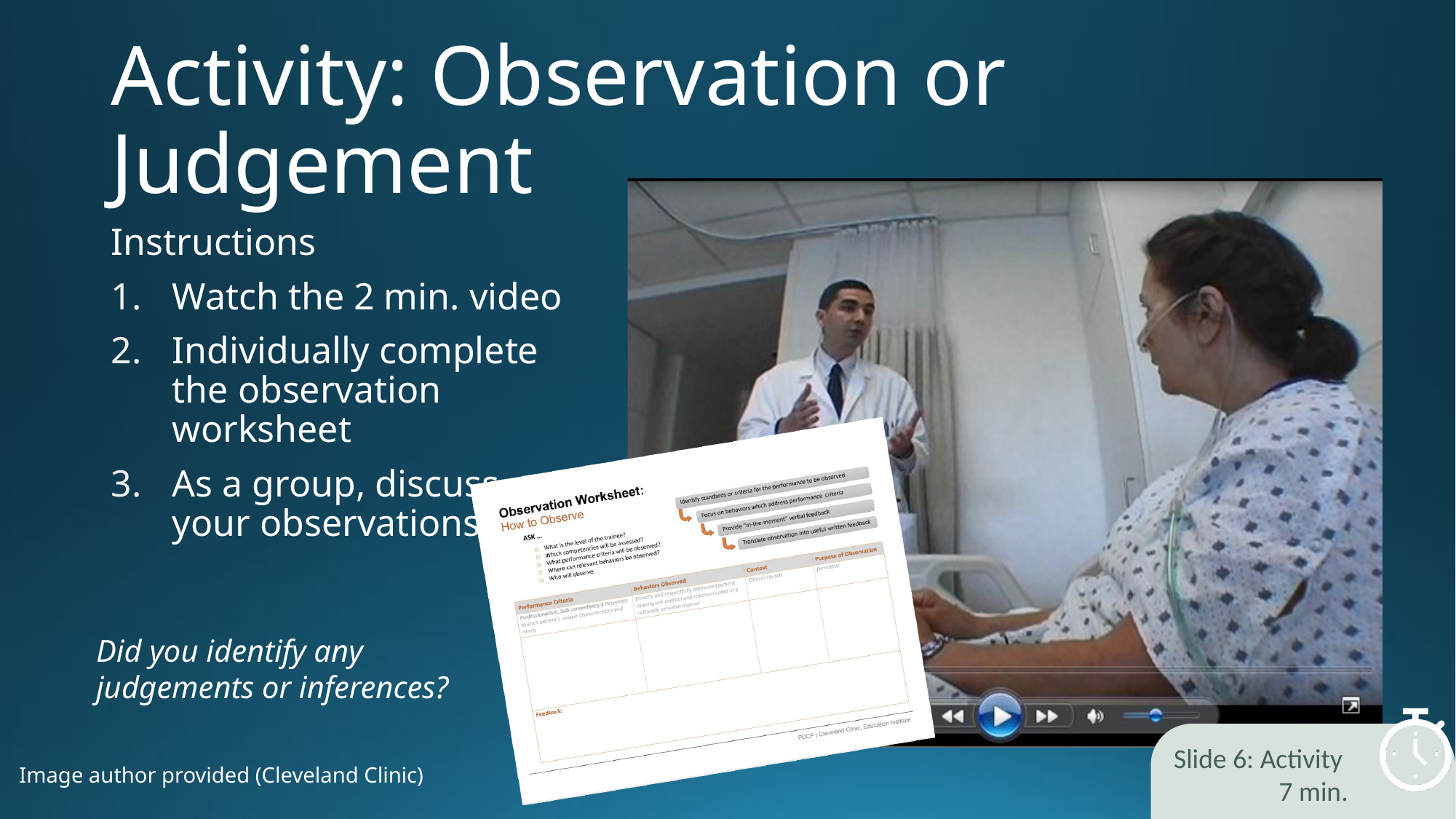

# Activity: Observation or Judgement
Instructions
Watch the 2 min. video
Individually complete the observation worksheet
As a group, discuss your observations
Did you identify any judgements or inferences?
Slide 6: Activity
 7 min.
Image author provided (Cleveland Clinic)

## Slide 9
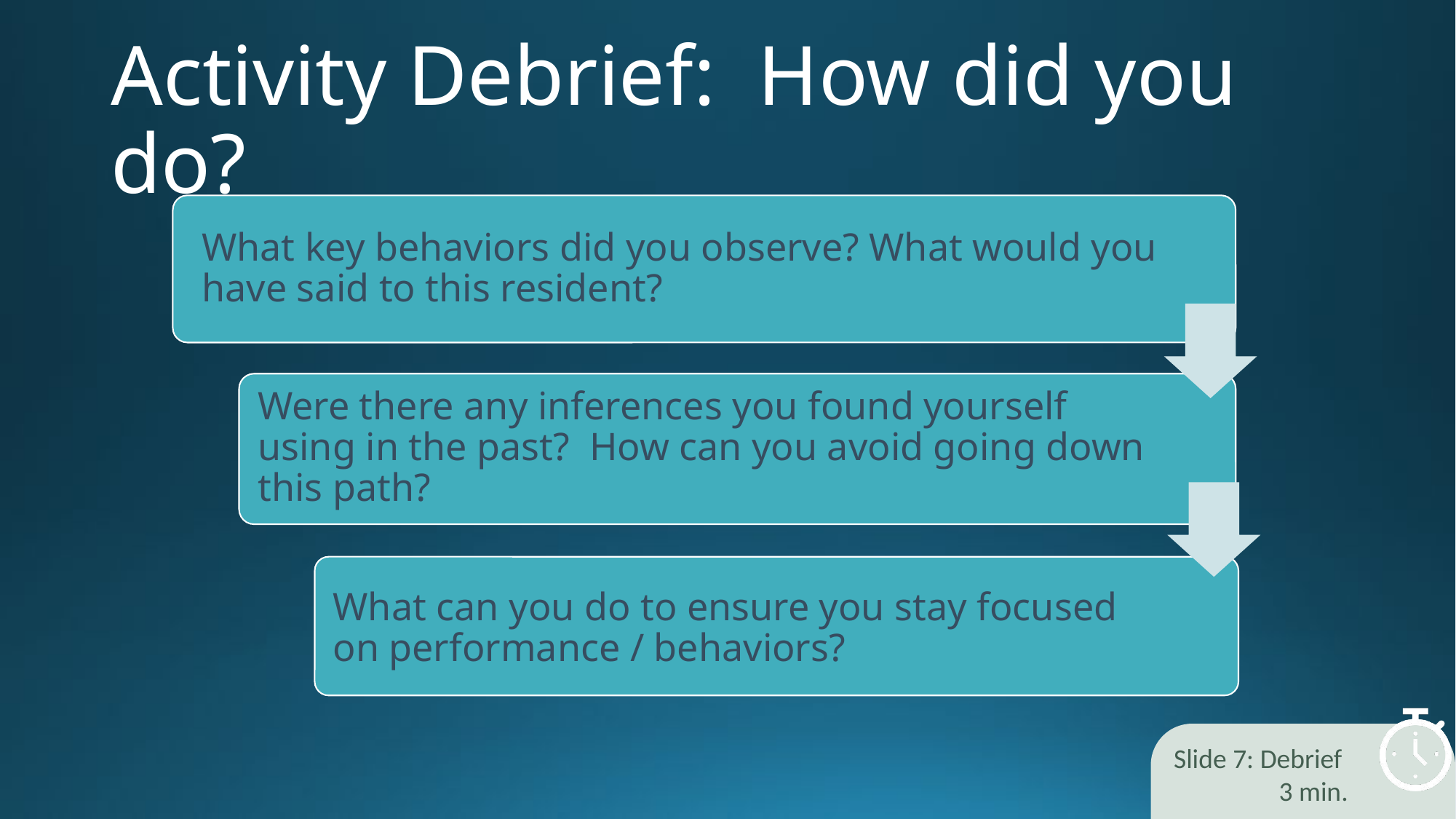

# Activity Debrief: How did you do?
What key behaviors did you observe? What would you have said to this resident?
Were there any inferences you found yourself using in the past? How can you avoid going down this path?
What can you do to ensure you stay focused on performance / behaviors?
Slide 7: Debrief
 3 min.

## Slide 10
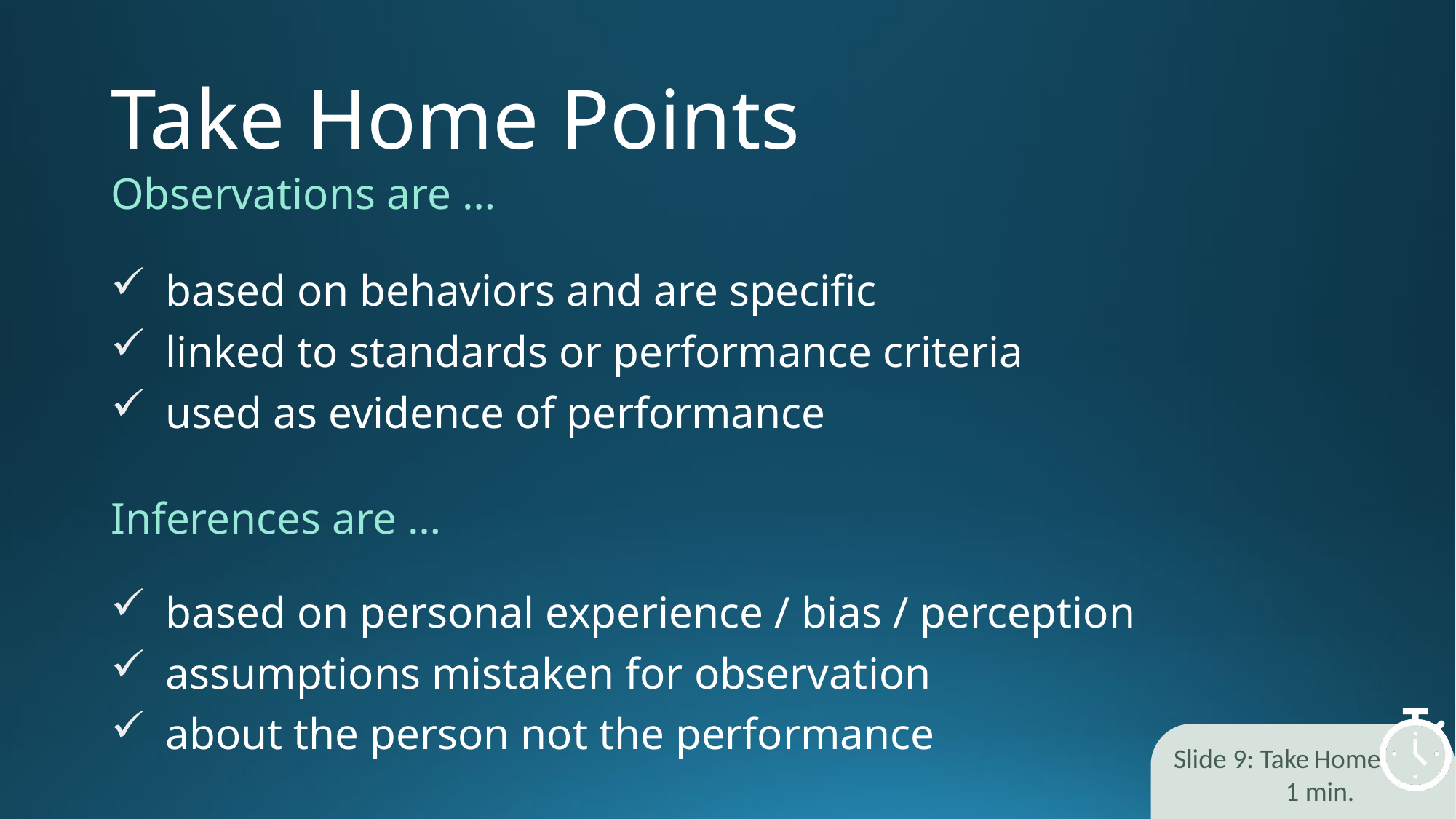

# Take Home Points
Observations are …
based on behaviors and are specific
linked to standards or performance criteria
used as evidence of performance
Inferences are …
based on personal experience / bias / perception
assumptions mistaken for observation
about the person not the performance
Slide 9: Take Home
 1 min.

## Slide 11
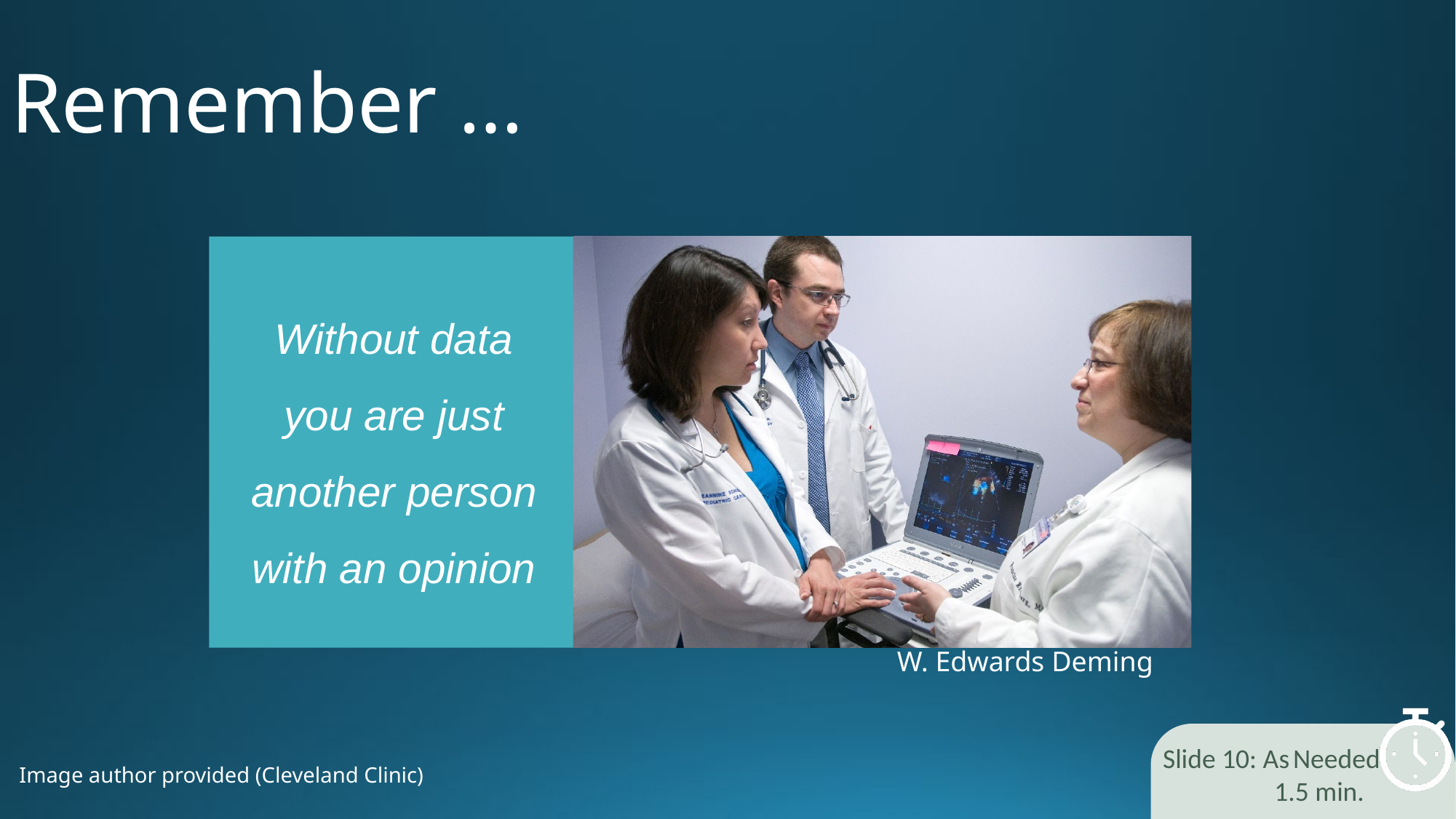

Remember …
Without data
you are just
another person
with an opinion
W. Edwards Deming
Slide 10: As Needed
 1.5 min.
Image author provided (Cleveland Clinic)

## Slide 12
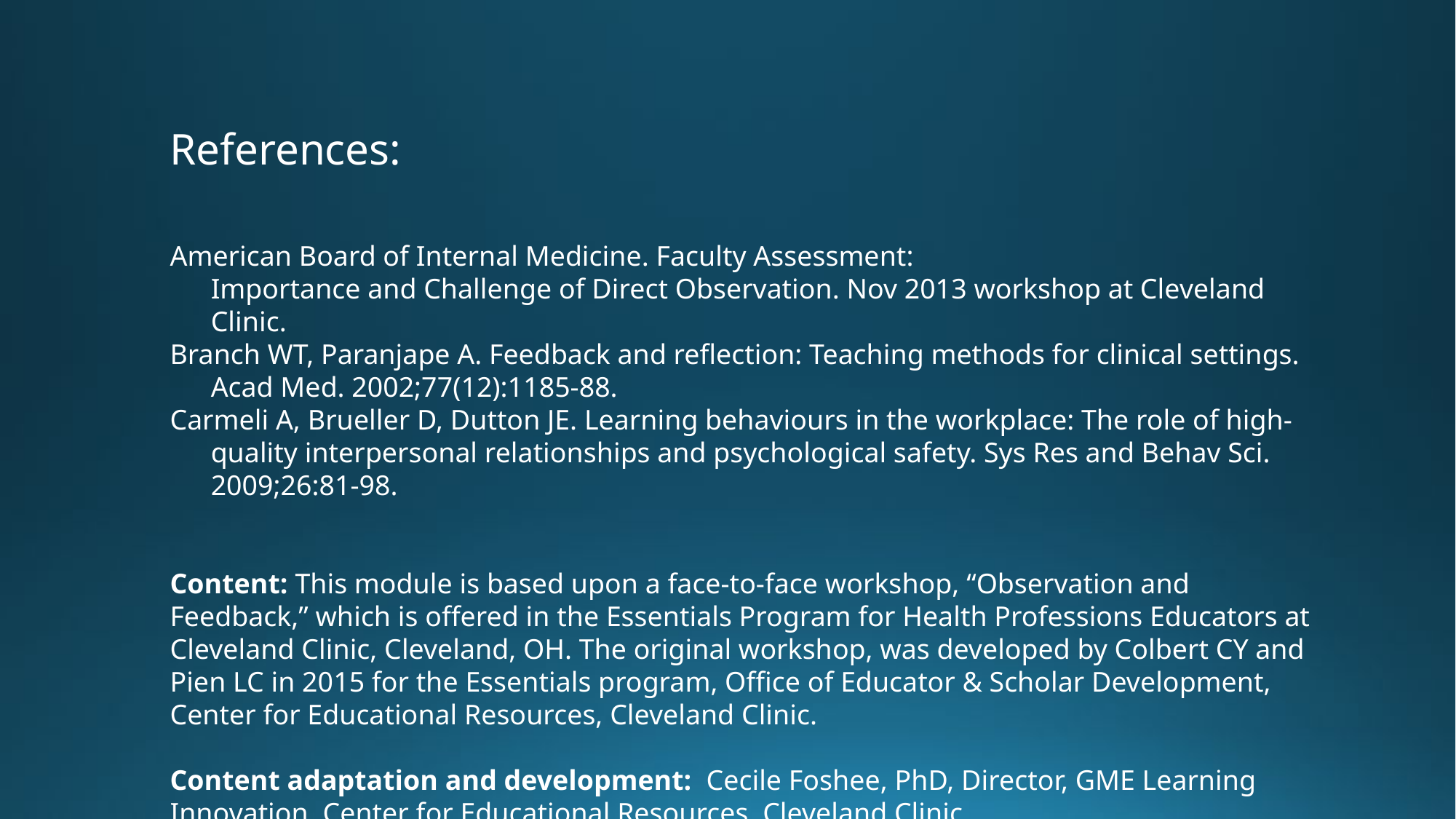

References:
American Board of Internal Medicine. Faculty Assessment:
Importance and Challenge of Direct Observation. Nov 2013 workshop at Cleveland Clinic.
Branch WT, Paranjape A. Feedback and reflection: Teaching methods for clinical settings. Acad Med. 2002;77(12):1185-88.
Carmeli A, Brueller D, Dutton JE. Learning behaviours in the workplace: The role of high-quality interpersonal relationships and psychological safety. Sys Res and Behav Sci. 2009;26:81-98.
Content: This module is based upon a face-to-face workshop, “Observation and Feedback,” which is offered in the Essentials Program for Health Professions Educators at Cleveland Clinic, Cleveland, OH. The original workshop, was developed by Colbert CY and Pien LC in 2015 for the Essentials program, Office of Educator & Scholar Development, Center for Educational Resources, Cleveland Clinic.
Content adaptation and development:  Cecile Foshee, PhD, Director, GME Learning Innovation, Center for Educational Resources, Cleveland Clinic
