# Supplementary material for: Got 15? Try Faculty Development on the Fly: A Snippets Workshop for Microlearning
Source: MedEdPORTAL. 2021 Jun 14;17:11161. doi: 10.15766/mep_2374-8265.11161 (PMC8200375; doi:10.15766/mep_2374-8265.11161)
Supplement: Supplementary file 1 — Snippet Presentation.pptxSession Plan.docxParticipant Email Message.docxSnippet Template.pptxCurated Materials Learning Environment.docxSmall-Group Instructions.docxExample of Completed Snippet.pptxWorkshop Evaluation.docx [file mep_2374-8265.11161-s001.zip › H. Workshop Evaluation.docx]

| Check 🡪 | | **Yes** | **No** | **Unsure** |
| --- | --- | --- | --- | --- |
| 1. I have participated in ‘snippet’-like (12-30 minutes) FD at my institution | |  |  |  |
| 1. I currently use a ‘snippet’-like FD strategy at my institution | |  |  |  |
| 1. Other’s perceive me as a competent Faculty Developer | |  |  |  |
| Check 🡪 | **0 /yr** (Not responsible) | **1-3 times/yr** | **4-6 times/yr** | **> 7 times/yr** |
| 1. I am responsible for creating or facilitating FD at my site/institution |  |  |  |  |
| 1. I plan to use snippets as a FD strategy |  |  |  |  |
| Check 🡪 | **Strongly DISagree** | **DIS**  **agree** | **Agree** | **Strongly Agree** |
| 1. I now have the skills to create FD snippets |  |  |  |  |
| 1. I plan to repurpose existing content as a FD snippet |  |  |  |  |
| 1. I plan to develop new content as a FD snippet |  |  |  |  |
| 1. I plan to access the FD snippets developed at this session |  |  |  |  |
| 1. I believe short (15-30 min) FD snippets can positively impact education practices |  |  |  |  |
| 1. Overall – this session was a valuable use of my time |  |  |  |  |
| 1. Are you interested in contributing to an international collection of FD snippets? |  |  |  |  |
| **If Yes** provide contact info: _________________________________________________________________ | | | | |

***Please provide a few comments so we can continue to improve our work!***

1. **Best thing about this session**
2. **1 thing you would urge us to change / do differently**
