# Supplementary material for: Got 15? Try Faculty Development on the Fly: A Snippets Workshop for Microlearning
Source: MedEdPORTAL. 2021 Jun 14;17:11161. doi: 10.15766/mep_2374-8265.11161 (PMC8200375; doi:10.15766/mep_2374-8265.11161)
Supplement: Supplementary file 1 — Snippet Presentation.pptxSession Plan.docxParticipant Email Message.docxSnippet Template.pptxCurated Materials Learning Environment.docxSmall-Group Instructions.docxExample of Completed Snippet.pptxWorkshop Evaluation.docx [file mep_2374-8265.11161-s001.zip › B. Session Plan.docx]

**Build a Snippet – Session Plan**

**Preparations:**

- Load PowerPoint presentation (Appendix A). We recommend editing to suit your topics and inserting appropriate images throughout the actual presentation for visual appeal
- Load example snippet (Appendix G). We recommend creating your own example snippet
- Set up room for small group work
- Distribute workshop materials
  - Clearly label each table with the appropriate topic.
  - Ensure each table has the activity instructions (Appendix F) turned over until ready to use.
  - The activity instructions should be revised to:
    a) reflect your own topics, and
    b) QR Codes and URLs should be updated to direct participants to your file locations.

**Session Plan**

| **Time** | **What to Show** | **What to Say** | **What to Do** |
| --- | --- | --- | --- |
| **1 min** |  | **Session Opening**  Slide 1  Facilitator Introductions | Important Note:  Before you begin, verify that all needed materials for the workshop have been loaded and tables have activity instructions |
| **2 min** |  | Slide 2  Facilitator should highlight the importance of faculty development | Refer to the speaker notes on the Power Point presentation for suggested script |

| **Time** | **What to Show** | **What to Say** | **What to Do** |
| --- | --- | --- | --- |
| **1 min** |  | Slide 3  Allow participants to come up with some words. Facilitator can write them on a board; have them call them out; or write them on a post-it note (share on chat for virtual) | Refer to the speaker notes on the Power Point presentation for suggested script |
| **1 min** |  | Slide 4  Review participant responses then highlight/emphasize those that match words on the slide | Refer to the speaker notes on the Power Point presentation for suggested script |
| **10 min** | Slide 5 Slide 6 | **Session Didactic**  Slides 5-9  Mini-Didactic on background of Snippet | (this portion is meant to demonstrate the use of the snippet model based on Mariam Bar-On)  Refer to the speaker notes on the Power Point presentation for suggested script |
|  |  Slide 7 |   Slide 8 |  Slide 9 |
| **Time** | **What to Show** | **What to Say** | **What to Do** |
| **4 min** |  | Slide 10  Be sure to explaining the structure of the template (slide 10) before moving on to instructions for the activity  Show completed sample snippet and highlight how the template is used in the example (Appendix G or your own sample) | Important Note:  Have your completed sample snippet loaded and ready to show. Remember that you are using this sample to illustrate a final product not teaching its content |
| **1 min** |  | Slide 11  Introduce the snippet building activity. Provide instructions  Be sure to orient everyone to the location of the materials  Highlight the timeline they should follow | Important Note:  Direct participants to location of activity materials (Appendix E updated with your details).  Assign small group facilitators |
| **40 min** | 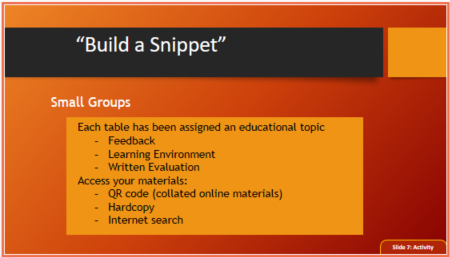 | **Session Activity**  Slide 12  Break into small groups depending on number of participants.  Ensure participants and small group facilitators are clear on their responsibilities  Ideally, each group should have a table/small group facilitator | Refer to the speaker notes on the Power Point presentation for suggested script  Important Note:  Workshop small group facilitators are responsible for helping/assisting in the development of a snippet for an assigned topic. Including helping participants navigate the needed activity materials. |

| **Time** | **What to Show** | **What to Say** | **What to Do** |
| --- | --- | --- | --- |
| **10 min** |  | **Session Close**  Slide 13  Snippet Debrief & Questions  Ask one group to share their snippet, this does not have to be completed snippet.  Ask about the group’s process (what worked, what didn’t work, successes, struggles, etc.)  Lead a large group discussion about the building process - have attendees share highlights, i.e.: Barriers, practicality, resources needed, opportunities, next steps. | Have a group share their created snippet  If the audience is large, group facilitators should identify the group(s) that should share their Snippet (the selection should be based on level of completion)  Depending on workshop setting, it may be necessary for group facilitators to save the group’s snippet to a thumb drive or upload to a shared folder |
| **3 min** |  | Slide 14  Take Home and Q&A  Elicit some key take home points from participants.  Remind them to shave their work | Attendees need to save/upload their completed snippets back to the folder where they obtained the template and materials (we recommend creating a folder labeled “finished snippets” then directing participants to save their work in that folder) |
| **2 min** |  | Slide 15  We recommend collecting feedback from your participants | Distribute evaluation forms (Appendix H or your own evaluation) |
