# Supplementary material for: Got 15? Try Faculty Development on the Fly: A Snippets Workshop for Microlearning
Source: MedEdPORTAL. 2021 Jun 14;17:11161. doi: 10.15766/mep_2374-8265.11161 (PMC8200375; doi:10.15766/mep_2374-8265.11161)
Supplement: Supplementary file 1 — Snippet Presentation.pptxSession Plan.docxParticipant Email Message.docxSnippet Template.pptxCurated Materials Learning Environment.docxSmall-Group Instructions.docxExample of Completed Snippet.pptxWorkshop Evaluation.docx [file mep_2374-8265.11161-s001.zip › Educational_Summary_Report_11161.pdf]

MedEdPORTAL® The AAMC Journal of Teaching and Learning Resources

Original Publication Open Access

Got 15? Try Faculty Development on the Fly: A Snippets Workshop for Microlearning

Carrie Bowler, EdD*, Cecile Foshee, PhD, Faye Haggar, EdD, Deborah Simpson, PhD, Clara Schroedl, MD, Heather Billings, PhD

*Corresponding author: bowler.carrie@mayo.edu

Abstract

Introduction: Faculty development (FD) is an element critical to the professional growth of medical educators and a necessary component in developing effective educators. FD offerings are prevalent across academic institutions; however, faculty report they are unable to participate in these initiatives due to time limitations and competing priorities. The snippet FD approach can address these concerns but requires training for FD providers to be effectively used. **Methods:** This snippet train-the-trainer workshop was presented to approximately 310 physician and nonphysician medical educators at a national medical education conference. The session incorporated multiple teaching modalities (e.g., lecture, demonstrations, structured small-group snippet development groups, and large-group debriefs). A 14-item Likert-scale survey was used to obtain participant evaluations. Narrative feedback was collected using constructed response items. **Results:** Ninety-five percent of respondents (125 of 132) planned to use snippets as an FD strategy at least once per year, with 38% (50 of 132) noting they planned to use snippets at least four times per year. Respondents indicated that FD snippets could positively impact educational practices (94%) and that the session was a valuable use of their time (94%), as well as expressing interest in a snippet repository (90%). **Discussion:** A brief FD train-the-trainer workshop for snippets can successfully prepare FD providers to create and use this approach.

Keywords
Workshop, Train the Trainer, Snippets, Microlearning, Faculty Development, Clinical Teaching/Bedside Teaching

Educational Objectives

By the end of this activity, learners will be able to:

1. Define the key components of a faculty development (FD) snippet.
2. Explain how multimedia principles, cognitive load theory, and communities of practice contribute to the development of a snippet.
3. Develop an FD snippet that can satisfy ACGME requirements.

Introduction

Most medical educators recognize the need for and importance of faculty development (FD), yet competing demands prevent many from taking advantage of traditional offerings at their institutions.[1,2] Time is the rarest commodity in any field, but it is particularly scarce in health care, where faculty must balance patient care with educational/professional growth efforts.[3] There has been tremendous work accomplished in the FD field, from conceptual frameworks to specific strategies.[4-7] We build upon this work by operationalizing an FD model that minimizes time spent creating content, mitigates cognitive load on faculty, and maximizes access to relevant and high-quality development materials and activities.

The inspiration for our work stemmed from our own experiences as faculty developers, the emerging communities within which FD is occurring, and Bar-on and Konopasek's[8] snippets model for FD. The essence of their work is focused attention on a single objective and careful alignment of the content and the learning activity to achieve the desired learning outcome. The most important component of this model is its emphasis on developing content around a single topic, with a single objective. To this end, we followed Bar-on and Konopasek's 10-slide PowerPoint approach[8] for the development of our FD workshop. Snippets offer a structured approach for delivering instruction in 15- to 20-minute interactive learning experiences. It is worth noting

Citation:
Bowler C, Foshee C, Haggar F, Simpson D, Schroedl C, Billings H. Got 15? Try Faculty Development on the Fly: a snippets workshop for microlearning. *MedEdPORTAL*. 2021;17:11161.
https://doi.org/10.15766/mep_2374-8265.11161

Copyright © 2021 Bowler et al. This is an open-access publication distributed under the terms of the Creative Commons Attribution license.

that a 15-minute presentation focusing on a single objective can be achieved without the use of a structured template but may require more knowledge, skills, and visuals to reinforce learning.

With any brief educational intervention, it is imperative to carefully consider what aspects of the presentation impact learning. This is where cognitive load theory[9] and multimedia principles[10,11] come into play. Cognitive load aims to explain how people process information for learning. There are three types of cognitive load: intrinsic, extrinsic, and germane.[9] Intrinsic load is imposed by the subject being learned. Extrinsic load is imposed by the way in which the instruction is delivered. Germane load refers to the mental resources required to process information into schemas. Germane load is thought to be negatively impacted by unnecessary mental demands. Thus, one should aim to decrease cognitive load by reducing extrinsic load and presenting information in ways that promote the development of information schemas or the conceptualization of ideas.[9] We used the following strategies within our presentation to mitigate cognitive load: the segmenting principle, where information was deliberately chunked into small units of explanation focusing on the what, why, and how of snippets; the coherence principle, where extraneous information was excluded from the didactic; and the redundancy principle, where our verbalization of slides enhanced rather than repeated text-based information displayed on the screen.[10,11]

While FD programs and resources were prevalent across our institutions, we noted that faculty members at our respective institutions were unable to engage in and/or attend these initiatives due to time limitations and other competing priorities. We recognized the need to format our FD opportunities to address these constraints. With these models, theories, and principles in mind, we set out to create a meaningful workshop for faculty developers. Our primary goal was to equip those responsible for providing FD with the skills and tools necessary to develop microlearning (snippets) at their own institutions. Based on a review of existing *MedEdPORTAL* FD publications, we contribute a proven approach to creating FD materials, rather than topic-specific FD (e.g., systems-based practice,[12] I-PASS Handoff Curriculum,[13] educator identity formation[14]). Here, we outline our process in detail and offer a robust sample of ready-made materials that a broad range of medical education professionals can use to create succinct, timely, and relevant FD. For the purposes of these workshops, we have limited the scope of FD topics to verbal feedback, learning environment, and written evaluation; however, the snippet model is relevant and feasible for the creation of FD materials related to other FD topic areas. This publication adds to the growing collection of FD resources in *MedEdPORTAL* specifically relevant for those seeking ways to create, facilitate, and document FD group activities.

Methods

Our workshop—Faculty Development on the Fly—was implemented at the 2020 national ACGME conference, which was attended by over 5,000 physician and nonphysician medical educators from across the world. Our workshop was developed so that anyone from the novice to the experienced medical educator could walk away with the knowledge, skills, and abilities to implement the snippet FD strategy at their own institution. As workshop facilitators, we were familiar with the FD practices and strategies used within medical education. The workshop was modified for this venue based on previous workshops facilitated at the AAMC's 2019 Central Group on Education Affairs Spring Conference and the 2019 Generalists in Medical Education Annual Conference.

Curricular Design of the Workshop

We designed a hands-on workshop that defined the concept of snippets and offered the audience an active learning experience to solidify their understanding of the concept and equip them with the skills to implement the FD strategy. Furthermore, we wanted to model the snippet format as part of the workshop process. We developed our workshop PowerPoint presentation (Appendix A) following the snippet format and created a session plan (Appendix B) to achieve these goals. Our snippet format was consistent in structure, with the timing extended beyond the 15-20 minutes, as the snippet was annotated for use in this train-the-trainer workshop.

Logistics of the Workshop

Faculty Development on the Fly was facilitated as a 75-minute workshop. All registered participants were given detailed instructions via email (Appendix C) prior to the workshop. Instructions included directions to access the workshop materials from the cloud and a recommendation to bring a personal device for content development. At the venue, we requested that the room be set up with round tables in order to facilitate small-group activity and engagement. Each table was clearly marked with one of three topics (verbal feedback, learning environment, or written evaluation). As participants entered the room, they were able to self-select a table based on their topic of interest.

Workshop kickoff—assessing prior knowledge and snippet introduction: The first 20 minutes were devoted to establishing the need for FD and illustrating how snippets can address this

Copyright © 2021 Bowler et al. This is an open-access publication distributed under the terms of the Creative Commons Attribution license.

need. More specifically, we started with a quick introduction of facilitators (5 minutes) and immediately launched into a 15-minute mini-didactic that defined and linked snippets to learning theories. We first asked the audience to reflect on the evolving needs of FD, followed by a facilitator-led debrief. Next, we introduced the snippets concept by engaging the audience in a think-pair-share activity. After setting the stage and hearing from the audience, we moved on to examining how snippets were constructed. The didactic was intentionally designed to follow the 10-slide PowerPoint model introduced by Bar-on and Konopasek.[8] Table 1 outlines the PowerPoint slide template used.

Workshop activity—building the snippet: To enhance the efficacy of our workshop, we intentionally limited the scope of FD topics to verbal feedback, learning environment, and written evaluation, important FD topics highlighted in the ACGME Common Program Requirements.[15] That said, the snippet model is suitable for the creation of FD materials related to other FD topic areas in a variety of settings, including live, virtual, and asynchronous self-paced learning.

To maximize the experiential component of the workshop and achieve our final objective, we allotted the next 40 minutes to the building of a snippet. During this time, participants developed their own snippet using a provided PowerPoint template (Appendix D) while working in small groups based on the FD topic assigned to their table: verbal feedback, learning environment, or written evaluation. We curated content materials, resources, and images relevant to the FD topics and housed all session materials within shared folders located on Google Drive. Appendix E includes an example of the curated materials for the learning environment topic. Instructions on how to access these materials were provided verbally at the time the activity started and in printed format available at each table (Appendix F). Because there were over 300 participants enrolled for our session workshop, we enlisted three additional colleagues to help facilitate the small-group activity. In total, we had eight facilitators who were responsible for checking in on the small groups in their assigned area to clarify expectations, answer questions, and provide input on the group's creation process for the snippet.

Table 1. Snippet PowerPoint Template

Slide No.	Slide Content
1	Title
2	Learning objective
3	Didactic
4	Didactic
5	Didactic
6	Activity instructions
7	Activity debrief
8	Take-home point
9	As needed
10	As needed

Workshop debrief—sharing a snippet and reflection on experience: The last 15 minutes included a showcase of one of our own snippets (Appendix G) designed and facilitated by author Cecile Foshee. Our intention was to illustrate a completed snippet product, demonstrate how to implement a snippet, and unpack the instructional design considerations used to maximize learning outcomes. We intentionally chose not to share the completed sample snippet prior to the activity as we wanted participants to be creative and not impose our own expectations for visual design. It is important to note that those planning to implement a train-the-trainer session using our approach may benefit from showing a finished example at the beginning of the workshop to help participants visualize the outcome.

Because the workshop was limited to 75 minutes in length, we were unable to have participants share examples of their created snippets as we had in prior workshops. However, this sharing can be achieved in 90 minutes and is a very validating and powerful activity. The snippet example was followed by a large-group discussion about building a snippet activity. The discussion also provided the opportunity for participants to ask questions and to share their experiences and lessons learned. As an added benefit to the participants, we saved all finished snippets to the Google Drive and shared links to the folder postsession with those who provided their emails. All participants who were interested were able to access a newly created repository of snippets. The Google Drive account we used did not include analytics, thus preventing us from tracking whether participants took advantage of this resource.

Evaluation Strategy and Instrumentation

We used the last 2 minutes to administer an optional session evaluation. We asked participants to complete the evaluation in paper form (Appendix H). The evaluation consisted of 14 questions that included selected response and constructed response items; Likert-scale items were rated on a 4-point scale (1 = *strongly agree,* 4 = *strongly disagree*). The first four questions aimed to gain an understanding of participants' background with FD and exposure to the snippet model. The subsequent seven items were designed to examine participants' competence, their intent to use the snippets FD strategy, and the overall session value. The next question inquired about participants' interest in contributing to an international repository of snippets. Finally, participants were asked to provide narrative

Copyright © 2021 Bowler et al. This is an open-access publication distributed under the terms of the Creative Commons Attribution license.

feedback with the following prompts: “The best thing about this session was…” and “One thing you would urge us to change/do differently is….” Using an iterative-grounded theory approach, one of the authors analyzed the quantitative and qualitative data collected to identify themes, with confirmation review by all authors.

Results

The ACGME reported that 310 individuals registered for our session, yet as is typical of large conferences, attendance was loosely monitored, being only limited by room capacity (300). As our room remained almost at capacity throughout the session, we used 300 as a conservative denominator. We collected 132 forms for a 43% response rate (132 of 310) to our workshop evaluation. We learned that less than 23% of respondents (30 of 132) had participated in snippet-like FD at their institution and that over 67% (89 of 132) had responsibility for creating or facilitating FD at their site/institution at least one time per year. However, only 17% (23 of 132) reported that they currently used a snippet-like FD strategy. At the conclusion of our workshop, 95% of respondents (125 of 132) planned to use snippets as an FD strategy at least one time per year, and 38% (50 of 132) were planning to use snippets at least four times per year. Table 2 highlights the participants’ snippet-specific FD competence, intended use, and overall perception regarding snippets. Ninety-four percent of respondents (124 of 132) agreed or strongly agreed that FD snippets could positively impact educational practices and agreed or strongly agreed that the session was a valuable use of their time, while 90% (119 of 132) agreed or strongly agreed they were interested in contributing to an international collection of FD snippets.

Over 175 narrative comments were provided by respondents and analyzed for themes. There were 102 comments related to the item about identifying the best thing about the session and 75 comments related to the item asking for improvements. While these were separate questions, some respondents included best-thing comments in the opportunities-to-improve response and vice versa. When multiple points were made by the same respondent, those responses were coded separately. Table 3 highlights the type of narrative responses we received; the table is organized by theme, with each theme showing the respective number of responses and overall percentages. The participants appeared to highly value the collaborative, interactive, hands-on nature of the session. Consistent with the results in Table 2, participants’ comments highlighted the practical nature of the workshop and the ease of development and use of the snippet model. Table 4 highlights the themes related to opportunities to improve the session. The feedback emphasized the need for additional instructions on the front end of the session and suggested walking through an example and/or showing the finished snippet example first. Feedback related to technical challenges concerned the conference facility’s WiFi capacity adversely affecting some participants’ ability to access the downloadable PowerPoint template. This was not an issue raised in the prior workshops as they had been facilitated in smaller venues. Overall, the participants were positive about the snippet FD model and the quality of the session.

Discussion

Our snippets workshop provided individuals who develop FD content with a practical train-the-trainer development framework. Aspiring and seasoned faculty developers walked away with the knowledge, skills, and abilities necessary to create brief and focused educational materials. As an outcome of this workshop, participants engaged in the development of a total of 32 snippets designed to be facilitated within a 15- to 20-minute interactive FD session. Through this workshop, we demonstrated the concept of cocreation of knowledge and built upon existing scholarship in the field. The snippet model addresses the time constraints of both the creator and the consumer. For those interested in facilitating this workshop, we provide the following reflection on important aspects associated with its development and delivery.

Table 2. Session Participants’ Evaluation Responses ($N = 132$)

Session Evaluation Items	Strongly Disagree	Disagree	Agree	Strongly Agree
I now have the skills to create FD snippets.	2%	6%	64%	27%
I plan to repurpose existing content as an FD snippet.	2%	9%	61%	28%
I plan to develop new content as an FD snippet.	2%	7%	59%	32%
I believe short (15-30 minutes) FD snippets can positively impact education practices.	2%	5%	65%	29%
Overall, this session was a valuable use of my time.	2%	3%	42%	52%
Are you interested in contributing to an international collection of FD snippets?	3%	7%	45%	45%

Abbreviation: FD, faculty development.

Copyright © 2021 Bowler et al. This is an open-access publication distributed under the terms of the Creative Commons Attribution license.

Table 3. Themes From Participants' Narrative Responses Addressing the Session's Best Features ($N = 102$)

Theme	Supporting Responses	No. of Responses	%
Collaborative	"Working with colleagues and exchanging ideas."<br>"Collaborative hands-on group work."	23	21
Practical tools and examples for implementation	"Concrete and easily adoptable tools for practical use."<br>"Framework to keep things concise, advice for adding to department meetings."	20	18
Active learning	"Actually using the technique and not just seeing a PowerPoint."<br>"Allowed for interaction of multiple persons who provided excellent ideas for faculty development."	19	17
Practical and feasible	"Made me realize I have the time and ability to do this."<br>"Understanding how easy/the way to create a useful tool."	15	14
Success	"One of the best and useful workshops I have attended in years."<br>"Well done—excellent topic with illustrative examples."<br>"It was a very informative and creative session. Great resources we can all use."	9	8

First, our goal was to develop an interactive workshop that resulted in equipping faculty developers with the resources and tools to generate snippets from preexisting FD materials. We were successful in achieving this goal as the evaluation data speak to the participants valuing the collaborative and interactive nature of the workshop as they cocreated content. We do caution that the quality of the snippets created during the workshop should be vetted prior to their being utilized.

Second, the active learning and experiential design was highly valued by participants. The workshop requires time for preparation and relies on technology to assist with this design. We facilitated the workshop at a national conference with over 300 registered participants. We used a shared Google Drive folder to house all the workshop materials. Registered participants were emailed instructions for accessing workshop materials and advised to bring a personal device for content development. During the session, we also provided verbal and written instructions for how to access the materials. However, several participants joined the workshop without having accessed the content in advance or bringing a device to access the materials or create content during the session. Thus, it is important to provide time during the workshop for all materials to be accessed, to pair up those who do not have a device with a participant who does, and to ensure the internet bandwidth is robust enough to handle that much connectivity. Furthermore, it is important to clearly articulate the interactive nature of the workshop and offer the opportunity for attendees to leave if participating in small-group activities does not meet their expectations. We recommend limiting the size of the workshop to that which is manageable for the facilitators and not allowing walk-ins in order to mitigate some of the issues we faced with this large an audience.

Third, we recommend sharing an example of the end result before the activity and providing clear instruction on the steps necessary to achieve this result early on. We designed the workshop to reinforce the snippet model and to equip the participants with the knowledge and skills to develop their own snippet. We engaged the participants in developing their own snippet and concluded with sharing an example of a created snippet. As experienced snippet creators, we did not recognize that a preponderance of novice creators first needed to experience a snippet rather than using a snippet format to

Table 4. Themes From Participants' Narrative Responses Addressing Change Opportunities ($N = 75$)

Theme	Supporting Responses	No. of Responses	%
Instructions	"More upfront orientation; I was a little unclear about the process. It was a bit rushed. Maybe showing the example in the beginning would create clarity."<br>"The intro instructions were confusing; Instructions for table exercise… bit confusing."	21	28
Technical challenges	"Improved technical assistance (problem with bring up videos and having a download with snippet PowerPoint slide would be great)."<br>"Need better instructions on website/materials/PowerPoint presentation."	14	19
Examples	"A better use of time would have been to deliver a snippet to us first and then work on it."<br>"We spent a lot of time trying to figure out what we were supposed to do, and seeing an example might have helped us bridge that learning gap."	10	13
Snippet topics	"Maybe provide some topics that attendees could build off."<br>"Maybe simpler topic to build first one easier to find references."<br>"Was not easy to discuss how to shape the assigned table exercise with colleagues who have different ideas."	7	9

Copyright © 2021 Bowler et al. This is an open-access publication distributed under the terms of the Creative Commons Attribution license.

teach them about snippets. Furthermore, because there is a risk of participants being at different comfort levels and having varying abilities with technology, it is important to build in more time for instruction.

Our evaluation data speaks to the success of the workshop design, its collaborative nature, and the active learning principles that have been integrated. There are opportunities for improvement that have been highlighted in our summary points for development and delivery. We are excited to see the future implications of this workshop as it has potential to contribute to a shared repository of open-access content for FD, as well as the possibility of showing how the content is being used within institutions. There is also an opportunity to develop a system to vet the developed content for quality prior to its release. Lastly, the workshop model can be used in both a face-to-face setting and a virtual environment. A virtual model for creation and delivery will be important as the practice environment changes, to provide access to subject matter experts and address financial constraints imposed by traditional FD methods.

Limitations

While the snippet resources used in this workshop can be readily adapted to different audiences, faculty developers who plan to employ the workshop will need experience designing, using, and evaluating snippets along with strong workshop facilitation skills. Although there is no substitute for snippet and faculty experience, understanding can be obtained by reading the articles referenced in the workshop materials. The evaluation results are strong and positive, yet some may have concerns regarding the low response rate. Our decision to use 300 (room capacity) as the workshop denominator was a conservative and defensible approach. Despite having data about interest in contributing to an FD snippet collection, we have no longitudinal follow-up of participants except for anecdotal comments we have received as to the powerful influence of the workshop on individuals' FD practice.

Future Directions

We have offered this workshop to many faculty members in medical schools and professional society meetings and are confident that it translates well across geographical, cultural, and professional boundaries. We have effectively offered the same workshop online, taking advantage of small-group discussions in breakout rooms. In the long term, we seek to explore the feasibility of an online collection of FD snippets given that over 90% of our ACGME session responses (119 of 132) were interested in contributing to such a repository.

Appendices

A. Snippet Presentation.pptx
B. Session Plan.docx
C. Participant Email Message.docx
D. Snippet Template.pptx
E. Curated Materials Learning Environment.docx
F. Small-Group Instructions.docx
G. Example of Completed Snippet.pptx
H. Workshop Evaluation.docx

All appendices are peer reviewed as integral parts of the Original Publication.

Carrie Bowler, EdD: Assistant Professor, Laboratory Medicine and Pathology, and Program Manager Graduate Medical Education, Mayo Clinic College of Medicine and Science; ORCID: https://orcid.org/0000-0001-6690-3999

Cecile Foshee, PhD: Assistant Professor of Medicine, Cleveland Clinic Lerner College of Medicine of Case Western Reserve University; Director of GME Learning Innovation and Vice-Chair of the Office of Interprofessional Learning, Cleveland Clinic

Faye Haggar, EdD: Assistant Professor and Director, Educational Development and Academic Technology, Department of Anesthesiology, University of Nebraska Medical Center College of Medicine

Deborah Simpson, PhD: Director of Education and Academic Affairs Advocate, Aurora Health; Professor (Clinical Adjunct) of Family Medicine, Medical College of Wisconsin and University of Wisconsin School of Medicine and Public Health

Clara Schroedl, MD: Assistant Professor of Medicine (Pulmonary and Critical Care) and Medical Education, Northwestern University Feinberg School of Medicine; ORCID: https://orcid.org/0000-0003-3280-4997

Heather Billings, PhD: Assistant Professor, Medical Education, Mayo Clinic College of Medicine and Science; ORCID: https://orcid.org/0000-0001-5867-0929

Acknowledgments

The authors would like to acknowledge the following individuals for their efforts to facilitate workshop components: Karen Marcdante, MD, Cindy Gosse, MS, and Deborah Hagen Moe, MEd.

Disclosures

None to report.

Funding/Support

None to report.

Copyright © 2021 Bowler et al. This is an open-access publication distributed under the terms of the Creative Commons Attribution license.

Ethical Approval

Reported as not applicable.

References

1. Montero L, Triviño X, Sirhan M, Moore P, Leiva L. Barreras para la formación en docencia de los profesores de medicina: una aproximación cualitativa. *Rev Med Chil.* 2012;140(6):695-702. https://doi.org/10.4067/S0034-98872012000600001
2. Steinert Y, Macdonald ME, Boillat M, et al. Faculty development: if you build it, they will come. *Med Educ*. 2010;44(9):900-907. https://doi.org/10.1111/j.1365-2923.2010.03746.x
3. Skeff KM, Stratos GA, Mygdal WK, et al. Clinical teaching improvement: past and future for faculty development. *Fam Med.* 1997;29(4):252-257.
4. Steinert Y, Mann K, Centeno A, et al. A systematic review of faculty development initiatives designed to improve teaching effectiveness in medical education: BEME Guide no. 8. *Med Teach*. 2006;28(6):497-526. https://doi.org/10.1080/01421590600902976
5. Skeff KM, Stratos GA, Mygdal W, et al. Faculty development: a resource for clinical teachers. *J Gen Intern Med.* 1997;12(suppl 2):S56-S63. https://doi.org/10.1046/j.1525-1497.12.s2.8.x
6. Morris C, Swanwick T. From the workshop to the workplace: relocating faculty development in postgraduate medical education. *Med Teach.* 2018;40(6):622-626. https://doi.org/10.1080/0142159X.2018.1444269
7. de Carvalho-Filho MA, Tio RA, Steinert Y. Twelve tips for implementing a community of practice for faculty development. *Med Teach*. 2020;42(2):143-149. https://doi.org/10.1080/0142159X.2018.1552782
8. Bar-on ME, Konopasek L. Snippets: an innovative method for efficient, effective faculty development. *J Grad Med Educ*. 2014;6(2):207-210. https://doi.org/10.4300/JGME-D-13-00362.1
9. Sweller J. Cognitive load during problem solving: effects on learning. *Cogn Sci.* 1988;12(2):257-285. https://doi.org/10.1207/s15516709cog1202_4
10. Moreno R, Mayer RE. A learner-centered approach to multimedia explanations: deriving instructional design principles from cognitive theory. *Interact Multimed Electron J Comput Enhanc Learn.* 2000;2(2):12-20.
11. Mayer RE. Applying the science of learning: evidence-based principles for the design of multimedia instruction. *Am Psychol*. 2008;63(8):760-769. https://doi.org/10.1037/0003-066X.63.8.760
12. Mirza A. Faculty development on systems-based practice. *MedEdPORTAL*. 2014;10:9909. https://doi.org/10.15766/mep_2374-8265.9909
13. O'Toole J, Sectish T, Starmer AJ, et al; I-PASS Education Executive Committee. I-PASS Handoff Curriculum: faculty development resources. *MedEdPORTAL*. 2013;9:9540. https://doi.org/10.15766/mep_2374-8265.9540
14. O'Sullivan PS, Irby DM. Educator identity formation: a faculty development workshop. *MedEdPORTAL*. 2021;17:11070. https://doi.org/10.15766/mep_2374-8265.11070
15. Common Program Requirements. Accreditation Council for Graduate Medical Education. Accessed August 21, 2020. https://acgme.org/What-We-Do/Accreditation/Common-Program-Requirements

Received: November 24, 2020
Accepted: March 28, 2021
Published: June 14, 2021

Copyright © 2021 Bowler et al. This is an open-access publication distributed under the terms of the Creative Commons Attribution license.
